# Integrated Ecotoxicological Assessment of Metal Nitrates in *Lemna minor* Based on Growth Inhibition, Biochemical Responses, and Species Sensitivity Comparison

**DOI:** 10.3390/toxics14090742

**Published:** 2026-08-22

**Authors:** Adina-Daniela Iachimov-Datcu, Diana-Maria Istrate, Lorena-Maria Toboșaru, Georgiana-Gabriela Doczi, Costel-Cristian Bulancea, Diana-Larisa Roman, Bianca-Vanesa Agachi, Constantina-Bianca Vulpe

**Affiliations:** 1 Department of Biology, Faculty of Chemistry, Biology, Geography, West University of Timisoara, Pestalozzi 16, 300115 Timisoara, Romania; daniela.iachimov@e-uvt.ro (A.-D.I.-D.); diana.istrate02@e-uvt.ro (D.-M.I.); lorena.tobosaru03@e-uvt.ro (L.-M.T.); doczigeorgiana10@gmail.com (G.-G.D.); bulancea.cristi2027@gmail.com (C.-C.B.); diana.roman@e-uvt.ro (D.-L.R.); 2 One Health Initiative-Focused Multidisciplinary Biosciences Advanced Research Center, West University of Timisoara, Oituz 4, 300086 Timisoara, Romania; constantina.vulpe@e-uvt.ro; 3 Department of Scientific Research in Biology, Advanced Environmental Research Institute, West University of Timisoara, Oituz 4, 300086 Timisoara, Romania

**Keywords:** duckweed, EC_50_, E_r_C_50_, toxic metals, water contamination

## Abstract

Heavy metals in water are a global concern due to their persistence, toxicity, and bioaccumulation potential, highlighting the need to assess their effects on aquatic organisms. The ecotoxicity of eight potentially toxic metal nitrates (Cd (0.002 mg/L), Co (0.02 mg/L), Cr (0.02 mg/L), Cu (0.001 mg/L), Mn (0.04 mg/L), Ni (0.005 mg/L), Pb (0.002 mg/L), Zn (0.1 mg/L) up to 1000 mg/L for all metals) was assessed on the aquatic macrophyte *Lemna minor*, in a multilevel study. Growth responses, measured as frond number and average specific growth rate, were used to generate dose–response curves, but also to calculate EC_50_ (Median Effective Concentration) and E_r_C_50_ (50% Effect Concentration based on growth rate) values. Based on EC_50_, toxicity followed the order Cd > Co > Ni > Zn > Cu > Cr > Pb > Mn, while E_r_C_50_-based ranking was Cd > Ni > Co > Zn > Cu > Cr > Pb > Mn. Metals were classified as highly toxic (Cd, Ni; Co based on EC_50_), moderately toxic (Co based on E_r_C_50_, Zn based on EC_50_), slightly toxic (Cu, Cr; Zn based on E_r_C_50_, Pb based on EC_50_), or practically non-toxic (Pb based on E_r_C_50_, Mn). Biochemical responses regarding photosynthetic pigments, primary metabolites, and oxidative-stress-related enzymes were investigated, revealing physiological effects for most metals. Species sensitivity distribution-based comparison indicated that *L. minor* sensitivity varies across metals, ranging from typical to relatively low within the broader species sensitivity distribution. Although the toxicity of potentially toxic metals to *L. minor* has been extensively investigated, data on E_r_C_50_ values for several of the metals investigated in the present study remain limited, highlighting the need for further ecotoxicological research.

## 1. Introduction

Pollutants from water bodies have become a universal challenge due to their increasingly common presence in aquatic habitats [1]. These environments are increasingly impacted by anthropogenic pollution originating from various sources, such as mining and metal-processing industries, manufacturing activities, agricultural runoff, municipal wastewater or atmospheric deposition [2,3,4]. A wide range of substances, including various classes of chemical pollutants, have been investigated for their occurrence and ecological effects in water environments [5,6,7,8,9]. Heavy metals, an important category of pollutants, are among the major contaminants described as hazardous to human health [10], and at the level of the European Union several legislative instruments regulate their presence [11,12,13].

Heavy-metal contamination of water represents a significant global environmental concern due to their persistence, toxicity, and potential to bioaccumulate in aquatic ecosystems [14], highlighting the need for a comprehensive understanding of their effects to aquatic organisms. Ecotoxicity assessments regarding heavy-metal effects were accomplished for numerous types of aquatic organisms, also reflecting their movement through aquatic food webs. These include the plants *Myriophyllum aquaticum*, *Mentha aquatica*, *Ludwigina palustris* [15], *Neptunia oleracea* [16], and *Nasturtium officinale* [17], fish [18], macroinvertebrates [19,20,21], zooplankton [22,23], and algae [24,25,26]. There is a documented growing tendency of toxicity to living species: Ag (Silver) > Cu (Copper) > Zn (Zinc) > Ni (Nickel) > Pb (Lead) > Cd (Cadmium) > Cr (Chromium) > Sn (Tin) > Fe (Iron) > Mn (Manganese) > Al (Aluminum) [27]. In order to determine the ecotoxicological effects of potentially toxic metals from waters, bioassessments were also realized using *Lemna minor* (L.) Griffith 1851, commonly known as duckweed. This plant species is a standard aquatic macrophyte recommended for phytotoxicity testing in the Organization for Economic Co-operation and Development (OECD) Guideline no. 221—*Lemna* sp. Growth Inhibition Test [28]. *L. minor* is a fast-growing, floating freshwater plant species from the family Araceae, which is widely distributed worldwide [29] and is responsive to environmental contaminants [7,30]. It is a useful model for phytotoxicity assessment due to the fact that it is easy to cultivate, has well-known endpoints, and it reproduces quickly [31]. Also, as an aquatic primary producer, *L. minor* plays a central role in linking waterborne contaminants to ecosystem processes due to their position at the base of aquatic food webs.

Although the phytotoxicity of potentially toxic metals toward *L. minor* has been extensively investigated, differences in experimental protocols, metal speciation, exposure conditions, and evaluated endpoints complicate direct comparison among studies. The OECD Test Guideline 221 recommends the use of growth inhibition endpoints, including frond number and average specific growth rate, for deriving EC_50_ and E_r_C_50_ values, providing a standardized framework for toxicity assessment. However, the reported toxicity values available in the literature frequently originate from studies employing different experimental designs or focusing on individual endpoints, making consistent comparison of the relative phytotoxicity of different heavy metals challenging. Therefore, generating toxicity data for multiple compounds under identical experimental conditions provides a robust basis for comparative evaluation while minimizing methodological variability.

In the present study, eight metal nitrates were evaluated using a standardized OECD 221-based experimental approach under identical exposure conditions. Growth inhibition was assessed using both frond number and specific growth rate to determine EC_50_ and E_r_C_50_ values, respectively, complemented by comparative biochemical analyses and species sensitivity distribution (SSD)-based comparison. Furthermore, considering the extensive use of nitrate fertilizers in agricultural systems, nitrate represents a major contaminant associated with soil leaching and agricultural runoff, contributing to the contamination of aquatic environments [32,33]. Consequently, nitrate can become an important component of the exposure scenario for aquatic macrophytes, particularly in agricultural-impacted ecosystems. As nitrate availability is closely related to nutrient acquisition processes and may influence ion transport mechanisms and metal uptake dynamics in plants [34], the assessment of heavy-metal toxicity using nitrate salts provides environmentally relevant exposure conditions that better reflect potential contamination scenarios in freshwater systems [35]. This integrated approach provides directly comparable toxicity data for eight metal nitrates within a single experimental framework and supports a comprehensive evaluation of their relative phytotoxicity and ecological relevance.

The main objective of this work is to evaluate the ecotoxicological effects of eight metal nitrates (Cd(NO_3_)_2_, Co(NO_3_)_2_, Cr(NO_3_)_3_, Cu(NO_3_)_2_, Mn(NO_3_)_2_, Ni(NO_3_)_2_, Pb(NO_3_)_2_, and Zn(NO_3_)_2_) on the common aquatic macrophyte *L. minor*, providing a comprehensive assessment of their multilevel effects on the aquatic environment. Heavy-metal nitrates were employed because of their high water solubility and rapid dissociation, ensuring the availability of dissolved metal ions for toxicity assessment. Furthermore, nitrate is among the most widespread inorganic contaminants in freshwater systems, with concentrations reported between 1.7 and 137 mg/L in groundwater [36] and between 0 and 135.2 mg/L in wells and springs [37,38].

Since nitrate and potentially toxic metals frequently coexist in aquatic environments impacted by agricultural and industrial activities, investigating the effects of heavy-metal nitrates contributes to a better understanding of the ecological risks associated with dissolved metal contaminants. Based on the available knowledge regarding heavy-metal toxicity in aquatic organisms, it was hypothesized that exposure to different metal nitrates would induce concentration-dependent toxic effects in *L. minor*, affecting not only growth-related parameters but also biochemical responses associated with oxidative stress and cellular defense mechanisms.

## 2. Materials and Methods

### 2.1. Tested Metal Nitrate and Exposure Concentrations

For this study, eight compounds, containing potentially toxic metal nitrates and the same anion, were used: Cd(NO_3_)_2_ · 4H_2_O (purchased from Sigma Aldrich, St. Louis, MO, USA), Co(NO_3_)_2_ · 6H_2_O, Cr(NO_3_)_3_ · 9H_2_O, Cu(NO_3_)_2_ · 2.5H_2_O, Mn(NO_3_)_2_ · 4H_2_O, Ni(NO_3_)_2_ · 6H_2_O, Pb(NO_3_)_2_ anhydrous, and Zn(NO_3_)_2_ · 6H_2_O. (purchased from Carl Roth, Karlsruhe, Germany). Preliminary range-finding tests were conducted to identify an appropriate concentration range capable of producing a complete concentration–response curve.

The nominal test concentrations ranged from 0.002 to 1000 mg/L Cd(NO_3_)_2_ · 4H_2_O (equivalent to 0.0007–364.4 mg Cd/L), 0.02–1000 mg/L Co(NO_3_)_2_ · 6H_2_O (equivalent to 0.004–202.5 mg Co/L), 0.02–1000 mg/L Cr(NO_3_)_3_ · 9H_2_O (equivalent to 0.0026–129.87 mg Cr/L), 0.001–1000 mg/L Cu(NO_3_)_2_ · 2.5H_2_O (equivalent to 0.00027–273.2 mg Cu/L), 0.04–1000 mg/L Mn(NO_3_)_2_ · 4H_2_O (equivalent to 0.0088–218.87 mg Mn/L), 0.005–1000 mg/L Ni(NO_3_)_2_ · 6H_2_O (equivalent to 0.001–201.84 mg Ni/L), 0.002–1000 mg/L Pb(NO_3_)_2_ (equivalent to 0.00125–625.58 mg Pb/L), and 0.1–1000 mg/L Zn(NO_3_)_2_ · 6H_2_O (equivalent to 0.022–219.77 mg Zn/L).

The tested concentration ranges were selected to encompass environmentally relevant exposure levels while also extending to higher concentrations required to establish robust concentration–response relationships. Expressed as equivalent elemental metal concentrations, the lowest tested concentrations were within or close to concentrations reported for surface waters [39], whereas some of the higher concentrations may be representative of heavily polluted waters or extreme contamination scenarios [40,41].

### 2.2. Testing Organism and Toxicity Assays

Toxicity assays were performed using *L. minor* colonies, using an experimental protocol adapted from the OECD 221 Guideline, “*Lemna* sp. Growth Inhibition Test” (2006), a widely accepted and currently applied methodology for evaluating the toxicity of chemical substances to aquatic plants [42,43], enabling a multilevel assessment encompassing growth effects, biochemical responses, and SSD-based species sensitivity comparison. Unless otherwise specified in the following sections, the experimental procedures were conducted in accordance with OECD 221.

*L. minor* was obtained from a stock culture and acclimated under laboratory conditions for two weeks prior to the experiments. Colonies were cultivated in 30 L of Swedish Standard (SIS) *Lemna* growth medium, prepared according to OECD Guideline 221 (containing 0.0114 mg Zn/L) [44], in a 50 L aquarium under static conditions. During the acclimation period, plants were maintained under continuous illumination at approximately 7800 lux and a constant temperature of 22 ± 1 °C, regulated using an aquarium heater.

Next, ten fronds per replicate, arranged in three colonies (two colonies consisting of three fronds each and one colony consisting of four fronds), were transferred into 30 mL of the respective test solution contained in single-use polypropylene (PP) vessels, ensuring a solution depth of approximately 2–3 cm. The vessels were covered with perforated plastic film to minimize evaporation while allowing gas exchange. The exposure was conducted under controlled conditions, including continuous illumination (approximately 7900 lux, within the range recommended by OECD Guideline 221), an ambient temperature of 24 ± 2 °C, and static exposure for seven days. The test medium was adjusted to pH 6.5 ± 0.2 prior to the preparation of the test solutions. No additional pH measurements were performed during the exposure.

Two controls were also analyzed in the same conditions: the positive control (C+) was represented by a 0.5% ZnCl_2_ solution that was selected as a positive control due to its well-known and reproducible toxic effect on *L. minor*, and its use as a reference toxicant consistent with United States Environmental Protection Agency guidance and the Organization for Economic Co-operation and Development framework. The negative control (C−) was represented by culture media. All tests were conducted in three replicates.

The endpoints of this toxicity test were the average specific growth rate (GR, *μ(i − j*)) (calculated according to OECD 221, Equation (1)) and the relative final frond number (NF), expressed as a percentage of the negative control. Dose–response curves were constructed using these two endpoints to determine the EC_50_ based on relative final frond number (EC_50_) and the E_r_C_50_ based on average specific growth rate. Moreover, it is important to mention that every new frond was taken into consideration when it was visible in the colony. These endpoints were calculated based on the nominal test concentrations, as the exposure concentrations were not analytically verified during the experiment.

The calculated EC_50_ and E_r_C_50_ values were used for the category determination of the tested samples in accordance with the U.S. Environmental Protection Agency’s (EPA) Guidelines on aquatic ecotoxicity [45]. The classifying system proposed by the EPA is: very highly toxic (<0.1 mg/L), highly toxic (0.1–1 mg/L), moderately toxic (>1–10 mg/L), slightly toxic (>10–100 mg/L) and practically non-toxic (>100 mg/L). Although a European [46] aquatic hazard classification framework exists, it was not applied because it is based on short-term E_r_C_50_ exposure tests (typically 72–96 h) for aquatic plant toxicity, whereas this study used a 7-day *L. minor* assay (OECD 221), resulting in a longer exposure duration and a non-aligned endpoint; additionally, it provides only a single acute classification category for aquatic acute toxicity (Aquatic Acute 1), while the EPA system offers more differentiated EC_50_-based hazard bands better suited for interpretation of the generated data.

(1) $$\mu_{i-j}=\frac{{ln(N}_{j})-{ln(N}_{i})}{t}$$

where

- *Μ_i−j_*: Average specific growth rate from time i to j;
- *N_i_*: Measurement variable in the test or control vessel at time i;
- *N_j_*: Measurement variable in the test or control vessel at time j;
- *t*: Time period from i to j.

### 2.3. Biochemical Testing

To better understand the metal nitrate compound effects, the ecotoxicity assessment was continued with the determination of some biochemical indices, including oxidative-stress-related enzymes, some primary metabolites and photosynthetic pigments. Three nominal concentrations (0.001, 1 and 1000 mg/L) were initially selected to represent low-, elevated- and extreme-exposure scenarios, respectively. The lowest concentration corresponded to a low-level exposure, whereas the intermediate concentration represented an elevated contamination scenario. The highest concentration was included to represent an extreme-exposure scenario; however, it resulted in severe growth inhibition and insufficient fresh biomass for reliable biochemical quantification. This response was considered a genuine biological effect of the treatment rather than an experimental artifact, as the Growth Inhibition Test fulfilled the OECD 221 validity criteria, with a control doubling time of 1.73 days (i.e., below the maximum acceptable value of 2.5 days). Consequently, biochemical analyses were restricted to the 0.001 and 1 mg/L treatments, which provided sufficient biomass for robust comparative evaluation of biochemical responses across the tested metal nitrates.

The resulting colonies were separated into two batches for two different extracts. For each, fresh weight (FW) was determined by gently patting dry the *L. minor* colonies on an absorbent paper towel to remove excess surface medium, followed by weighing the plant material using an analytical balance with five decimal places (ABT220, Kern, KERN & Sohn GmbH, Balingen-Frommern, Germany).

Both types of extracts were realized by grinding the vegetal material using a tissue homogenizer and disperser (MiniBatch D-1, MICCRA GmbH, Heitersheim, Germany) either in 80% acetone or in phosphate buffer (pH 7). The homogenates were then centrifuged at 18,000 rpm (Mikro 22 R, Hettich, Andreas Hettich GmbH & Co. KG, Tuttlingen, Germany) for 15 min, and the resulting supernatants were collected for the analysis of biochemical parameters.

Two oxidative-stress-related enzymes were investigated, more specifically catalase and guaiacol peroxidase. Catalase (CAT) activity was determined according to the method described by A. K. Sinha (1972) [47], as previously applied in our earlier study [48]. The assay is based on the quantification of hydrogen peroxide remaining after incubation with the phosphate buffer extract for 1 and 3 min. The reaction mixture, comprising 40 µL of 0.2 M H_2_O_2_ and 60 µL of enzyme solution (phosphate buffer extract), was incubated for 1 and 3 min in separate experiments. After incubation, the solution was completed with 200 µL of 5% potassium dichromate in glacial acetic acid and was heated at 100 °C for 10 min. Residual hydrogen peroxide was determined through its reaction with potassium dichromate in acetic acid to form chromic acetate, which was measured spectrophotometrically at 570 nm using a microplate reader (Synergy H1, Agilent Technologies (formerly BioTek Instruments), Santa Clara, CA, USA) and quantified against a standard curve prepared using hydrogen peroxide and potassium dichromate in acetic acid. The enzymatic activity was calculated from the consumption of the substrate (H_2_O_2_) over a 2 min interval.

Guaiacol peroxidase (GPX) activity was assessed by preparing a reaction mixture consisting of 50 µL of 1% guaiacol, 50 µL 0.2 M hydrogen peroxide, 130 µL of phosphate buffer and 20 µLof phosphate buffer extract, in a 96-well microplate. GPX activity was kinetically determined, by measuring at 470 nm the formation of reaction product tetraguaiacol from the substrate guaiacol, by a microplate reader (Synergy H1, Agilent Technologies—formerly BioTek Instruments—Santa Clara, CA, USA).

A primary metabolite, represented by total soluble proteins (P), was assessed spectrophotometrically (Synergy H1, Agilent Technologies (formerly BioTek Instruments), Santa Clara, CA, USA) at 562 nm, using a bicinchoninic acid (BCA) protein assay kit (Pierce 170 TM BCA Protein Assay Kit, Thermo Scientific, Cambridge, UK). The quantification was performed according to the instructions included in the kit, using a standard curve with bovine serum albumin and BCA solution.

The reducing sugar concentration (RS), another primary metabolite, as well as photosynthetic pigments, represented by chlorophylls a (Chl a), b (Chl b), a + b (Chl a + b) and carotenoids (Car; xanthophylls + carotenes, x + c), were assessed according to the quantification protocols described in a previous study [48], using a microplate reader (Synergy H1, Agilent Technologies (formerly BioTek Instruments), Santa Clara, CA, USA). The reducing sugar concentrations were determined using a standard curve with glucose and 3,5-dinitrosalicylic acid (DNS) solution, while the photosynthetic pigments were quantified using the equations described by Lichtenthaler and Wellburn (1983) [49].

### 2.4. Estimation of Hazardous Concentrations

The ETX software (a program to calculate hazardous concentrations and fraction affected, based on normally distributed toxicity data, version 2.3.1) [50,51,52] was used to analyze species sensitivity distribution (SSD) in order to estimate the hazardous concentration of each metal nitrate for 5% (HC_5_) and 50% (HC_50_) of species in a community. HC_5_ represents the concentration expected to affect 5% of species, while HC_50_ corresponds to the concentration affecting 50% of species within the distribution. These parameters were estimated using a series of data for each tested compound which included the experimental EC_50_ and E_r_C_50_ values determined in this study for *L. minor*, as well as similar toxicological endpoints (EC_50_, LC_50_, LD_50_, etc.) from the literature. The literature data was identified using the U.S. Environmental Protection Agency’s (EPA) ECOTOX database [53,54], by extracting these endpoints for aquatic species (data was used as given from the database in median concentration standardized for mg/L).

### 2.5. Statistical Analysis

In order to accomplish the data processing, Microsoft Office Excel 365 was utilized. All the statistical assessments, including plotting the dose–response curves and other graphs, and calculating the EC_50_ and E_r_C_50_ values with confidence intervals for all compounds, were done with OriginPro software (OriginPro Version 2025, OriginLab Corporation, Northampton, MA, USA). The dose–response relationship was fitted using a non-linear logistic function, and EC_50_/E_r_C_50_ values were derived from the fitted curves. For the data normality testing, the Shapiro–Wilk W test was used. After analyzing the distribution, ANOVA analysis was conducted, followed by Tukey’s post hoc test to analyze variances. The differences were considered significant at *p* < 0.05.

## 3. Results and Discussion

### 3.1. Growth Inhibition Test Results

Both the number of fronds, a relevant endpoint for *L. minor* growth assessment, and the average specific growth rate, a standard growth parameter in the OECD 221 Guideline [28], showed concentration-dependent trends in response to increasing metal nitrate concentrations. This allowed the plotting of dose–response curves and the calculation of half-maximal effective concentrations. In the dose–response curves based on relative number of fronds (% of C−) and potentially toxic metal salt concentrations (Figures S1–S8), the 95% confidence intervals showed better model efficiency for Cd, Ni and Zn nitrate.

For the dose–response curves plotted with average specific growth rate and metal nitrate concentration (Figures S9–S16), the models showed narrower confidence intervals for Cd, Cr, Cu, Mn, and Ni nitrates. Among these, cadmium and nickel nitrates exhibited the most precise fits based on both the relative number of fronds and the average specific growth rate.

**Figure 1:**
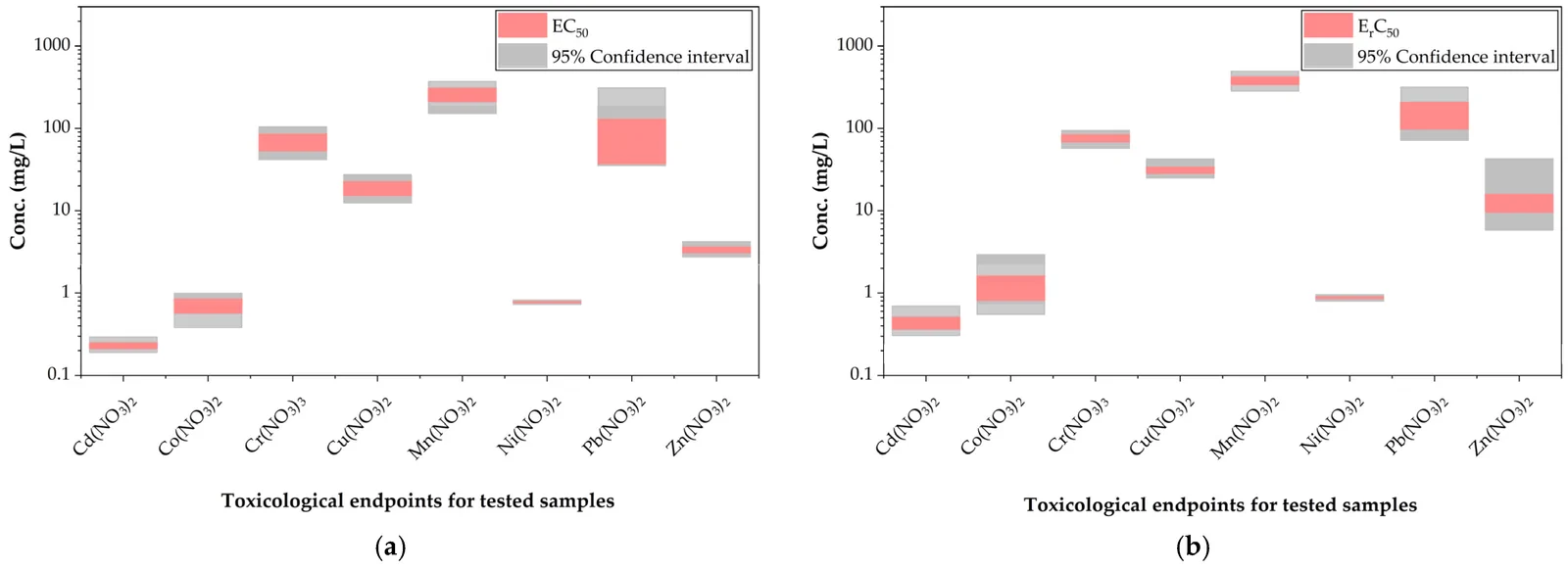
Toxicological endpoints for the tested samples: (**a**) EC_50_ and (**b**) E_r_C_50_ values. Gray areas represent 95% confidence intervals; red areas indicate the toxicological endpoint ± standard deviation.

The calculated EC_50_ and E_r_C_50_ values are shown Table 1 and represented in Figure 1, along with their 95% confidence intervals. The lowest values were observed for Cd, Co and Ni nitrates, the highest being observed for Cr, Mn and Pb nitrates. The order of toxicity based on EC_50_ values was determined to be Cd(NO_3_)_2_ > Co(NO_3_)_2_ > Ni(NO_3_)_2_ > Zn(NO_3_)_2_ > Cu(NO_3_)_2_ > Cr(NO_3_)_3_ > Pb(NO_3_)_2_ > Mn(NO_3_)_2_, and based on E_r_C_50_ values was Cd(NO_3_)_2_ > Ni(NO_3_)_2_ > Co(NO_3_)_2_ ()Zn(NO_3_)_2_ > Cu(NO_3_)_2_ > Cr(NO_3_)_3_ > Pb(NO_3_)_2_ > Mn(NO_3_)_2_. According to EPA aquatic ecotoxicity categories, Cd and Ni nitrates are highly toxic; Co(NO_3_)_2_ is highly toxic based on NF and moderately toxic based on GR; Zn(NO_3_)_2_ is moderately toxic based on NF and slightly toxic based on GR; Cu and Cr nitrates are slightly toxic; Pb(NO_3_)_2_ is slightly toxic based on NF and practically non-toxic based on GR; and Mn(NO_3_)_2_ is practically non-toxic.

The phytotoxic responses observed in the present study reflect the effects of the investigated heavy-metal nitrate salts under the applied experimental conditions. Because nitrate is an essential macronutrient and one of the principal nitrogen sources for higher plants, it may influence plant growth and nutrient uptake and therefore cannot be completely excluded as a contributing factor to the observed responses [55,56]. However, nitrate was already present in the SIS growth medium to ensure optimal growth of *L. minor*, whereas the additional nitrate introduced with the tested compounds reflects exposure conditions that may occur in nitrate-enriched aquatic environments. Consequently, although the contribution of nitrate cannot be completely excluded, previous studies have demonstrated that nitrate has low or no influence on metal toxicity [57]. Therefore, the observed toxicity is likely attributable predominantly to the heavy-metal component of the investigated nitrate salts.

The obtained EC_50_ and E_r_C_50_ values are environmentally relevant, particularly under contaminated conditions. When expressed as elemental concentrations, the EC_50_ values for Cd, Co, Ni, Cu, and Zn are within or below the concentration ranges reported for heavily contaminated waters, including acid mine drainage [40,41], while the EC_50_ for Zn is also lower than concentrations reported in some surface waters [39]. In contrast, the EC_50_ values for Cr, Mn, and Pb remain substantially higher than the reported environmental concentrations, suggesting a lower probability of acute phytotoxicity under the investigated exposure scenarios. Although these comparisons provide an indication of the potential environmental relevance of the observed toxicity, the present results were obtained under controlled laboratory conditions using dissolved metal nitrates. In natural aquatic systems, heavy-metal nitrates rapidly dissociate into metal and nitrate ions, which may coexist in the same aquatic environment. However, metal speciation and bioavailability are influenced by complexation with dissolved organic matter, adsorption to suspended particles and mineral surfaces, and water chemistry (e.g., pH and hardness), which can reduce the fraction of bioavailable metal and consequently alter phytotoxicity [58,59].

### 3.2. Biochemical Responses

**Figure 2:**
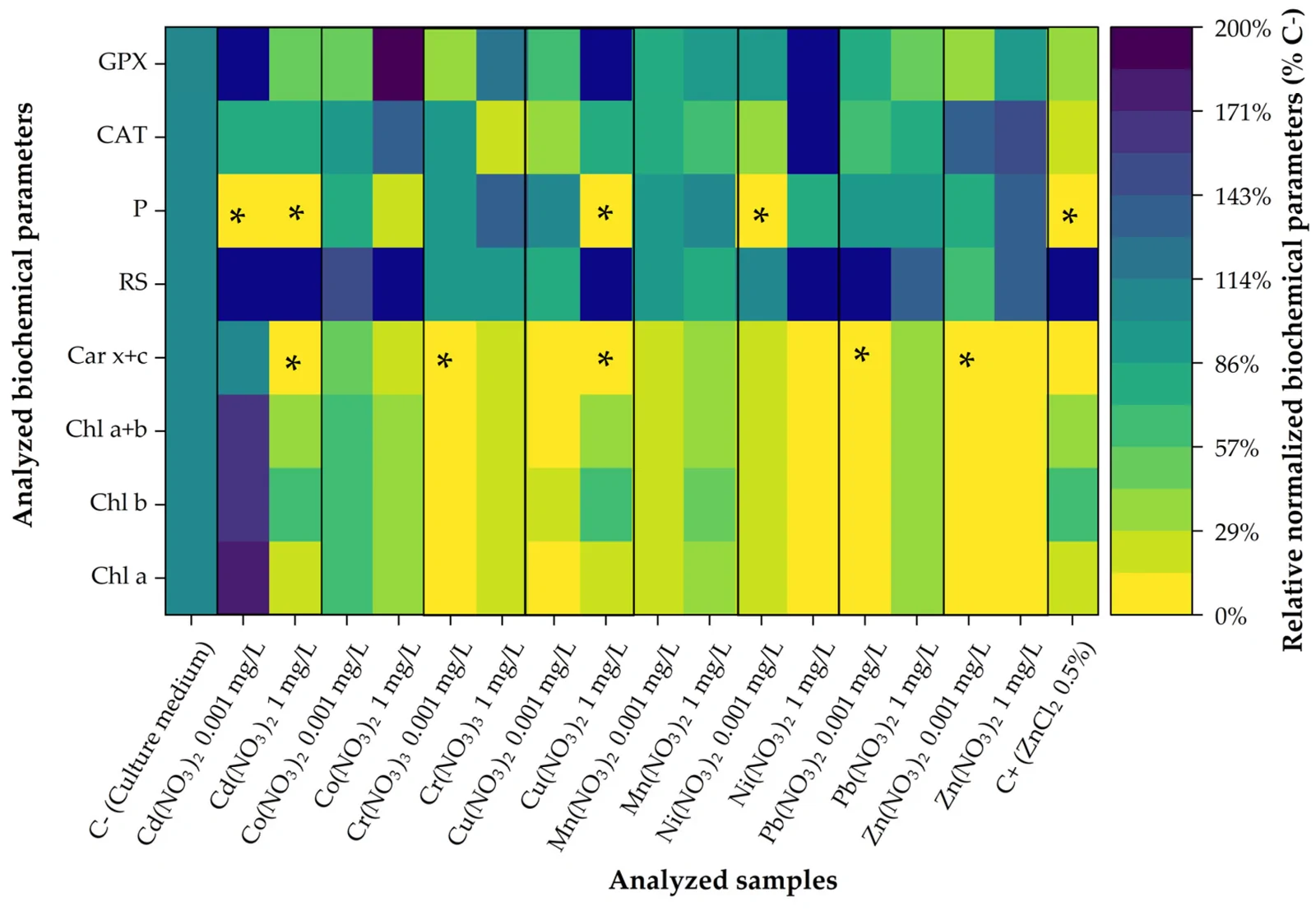
Comparative heatmap visualization showing the effects of metal nitrates on biochemical parameters across treatments, including photosynthetic pigments (chlorophyll a, chlorophyll b, total chlorophyll a + b, and carotenoids), primary metabolites (reducing sugars and total soluble proteins), and oxidative-stress-related enzymes (catalase and guaiacol peroxidase). The heatmap presents relative values derived from fresh-weight-normalized datasets. Data marked with * were below the limit of detection (LOD).

**Figure 3:**
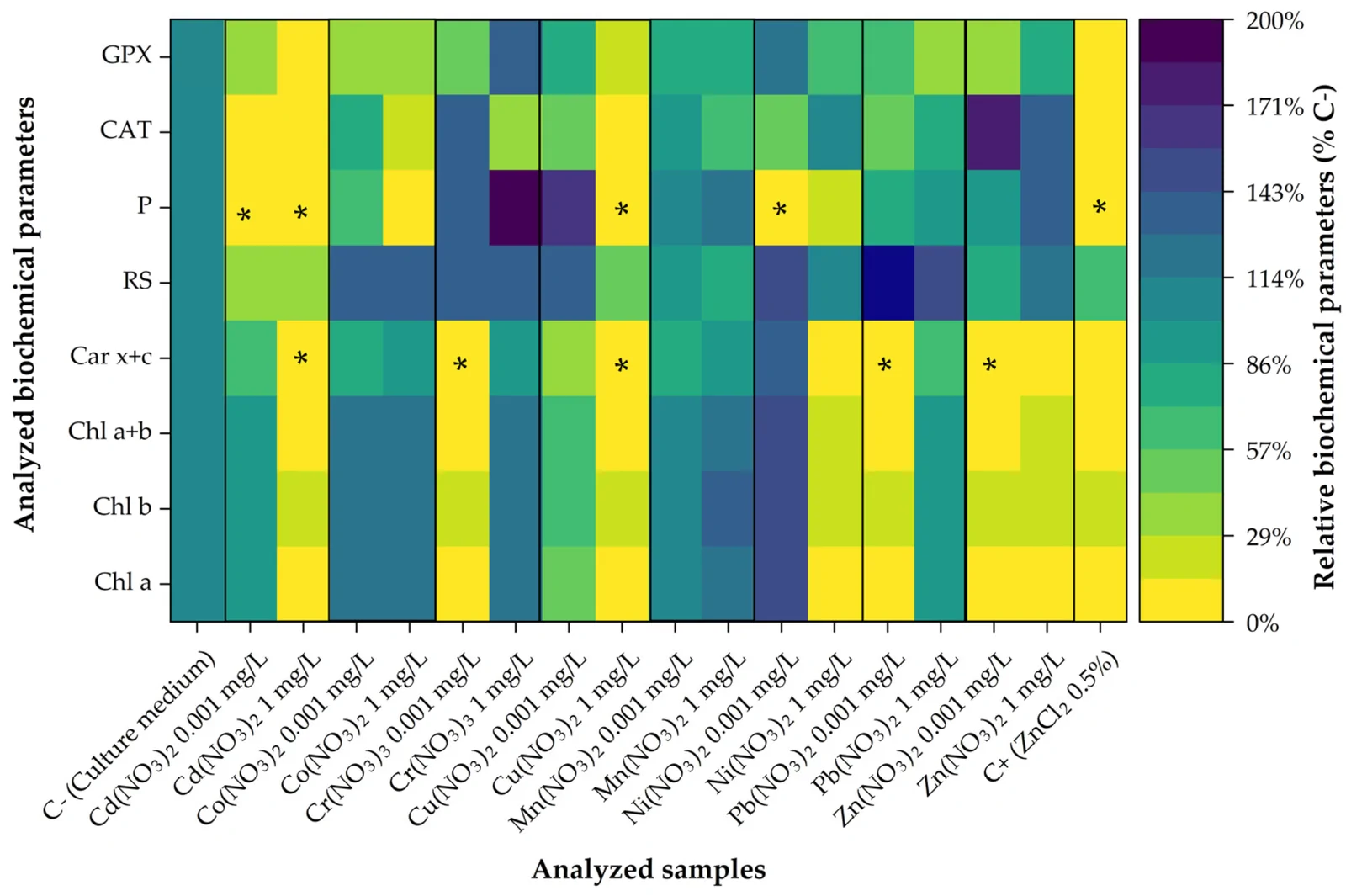
Comparative heatmap visualization showing the effects of metal nitrates on biochemical parameters across treatments, including photosynthetic pigments (chlorophyll a, chlorophyll b, total chlorophyll a + b, and carotenoids), primary metabolites (reducing sugars and total soluble proteins), and oxidative-stress-related enzymes (catalase and guaiacol peroxidase). The heatmap presents relative values derived from non-normalized datasets. Data marked with * were below the limit of detection (LOD).

The biochemical responses were expressed relative to the corresponding untreated control to enable comparison among treatments (Figure 2 and Figure 3).

Chlorophyll a, chlorophyll b, chlorophyll a + b, and carotenoids showed coordinated responses within treatments (Figure 2 and Figure 3). Co and Mn nitrates displayed a plateau-like pattern with relatively high pigment levels and minimal variation across tested concentrations, whereas Zn nitrate showed consistently low chlorophyll levels across all treatments, indicating strong suppression of pigment content. Cd, Cu and Ni nitrates showed a reduction in these pigments at 1 mg/L nominal concentration. Cr and Pb nitrates showed an increased content of pigments at the higher tested concentration. The fresh-weight-normalized data showed a generally reduced pigment concentration relative to the negative control for all tested nitrates, except for Cd(NO_3_)_2_ which had high levels at 0.001 mg/L. The statistical analysis (Supplementary Tables S2 and S3) showed that significant differences between the two tested concentrations of the same metal nitrate were observed only for Pb regarding chlorophyll a content, for Cr regarding chlorophyll b and a + b content, and for Ni regarding carotenoid contend, as not normalized data. The normalized data showed statically significant differences between the two tested concentrations of lead nitrate regarding chlorophyll a and a + b content. Significant differences among the tested metal nitrates were observed only between Cd and Zn nitrates as chlorophyll b content. The obtained data indicated that measured pigment levels depend on data expression and sample-related factors under stress conditions [60,61]. Literature evidence suggests that the effects of heavy metals on the photosynthetic apparatus are strongly dependent on their reactivity and concentration, where they can interfere with light harvesting, disrupt electron transport chains, and inhibit key photosynthetic enzymes [62,63]. In this context, reduced photosynthetic performance is mainly associated with decreased chlorophyll content. Heavy metals can impair chlorophyll through enhanced degradation, oxidative damage via reactive oxygen species, and inhibition of biosynthesis through displacement of Mg in the porphyrin ring [62]. Additionally, oxidative stress disrupts chloroplast structure and thylakoid organization, further limiting photosynthetic efficiency. Finally, key Calvin cycle enzymes may be inhibited through oxidative protein damage, reducing carbon fixation [62]. These mechanisms are reflected in the present results, where Zn, Cd, Cu, and Ni nitrates generally reduced chlorophyll and carotenoid contents, indicating impairment of the photosynthetic apparatus.

Reducing sugars were detected in all analyzed samples under both data representations (Figure 2 and Figure 3), though their distribution differed depending on whether values were fresh-weight-normalized or not. The non-normalized dataset showed high levels of reducing sugars compared to the negative control, except cadmium nitrate, although the normalized dataset showed high values for this nitrate as well. No significant differences were observed either between the two tested concentrations of the same metal nitrate or between the tested metal nitrates (Supplementary Tables S4 and S5). Elevated reducing sugar levels indicate an accumulation of soluble carbohydrates, which may reflect stress-related metabolic adjustment [64]. Reducing sugars are part of the soluble carbohydrate pool and reflect the balance between production, conversion, utilization, and storage under heavy-metal stress. Their decrease is associated with reduced carbohydrate synthesis and disruption of metabolic processes, leading to lower availability of soluble carbohydrates [62]. In contrast, increases may result from enhanced conversion of reserve carbohydrates into soluble forms, altered metabolic activity, and reduced utilization due to growth inhibition, leading to accumulation [65].

Total soluble protein content (Figure 2 and Figure 3) showed two effect patterns of the tested metal nitrates, except for Cd nitrate which showed low levels, below the detection limit for both tested nominal concentrations. Co and Cu nitrates showed a decrease in protein concentration with the increase in exposure concentration, while Cr, Mn, Ni, Pb and Zn nitrates induce an increase in protein concentration. These patterns were consistent between the fresh-weight-normalized and non-normalized datasets across all compounds. No significant differences were detected between the two tested concentrations of the same metal nitrate (Supplementary Tables S4 and S5). Between the tested metal nitrates, a significant difference was observed only between Ni(NO_3_)_2_ and Cr(NO_3_)_3_ for the non-normalized protein content, whereas no significant differences were found for the normalized values. Heavy metals can exert complex effects on protein content, involving both degradation and induced synthesis [62,66]. Decreases may result from protein oxidation, including covalent modification of proteins, peptide bond cleavage, oxidation of amino acids into protein carbonyls, and structural disruption via binding of some metals to sulfhydryl groups, followed by degradation via proteolytic systems. In contrast, increases may result from de novo synthesis of stress- and antioxidant-related proteins, including heat-shock proteins and other protective proteins, induced under metal exposure conditions [67,68,69].

Catalase activity (Figure 2 and Figure 3) showed, as well, different patterns under metal nitrate exposure. Cd nitrate showed low catalase activity at both tested concentrations, while the other compounds showed different effects. Co, Cr, Cu, Mn and Zn nitrates showed a decrease in catalase activity with increasing concentration, while Ni and Pb nitrates induced higher activity at the higher concentration. Due to fresh weight normalization, some patterns changed, such as in the case of cobalt, copper and zinc nitrates. Significant differences between the two tested concentrations were observed only for Cr(NO_3_)_3_ and Ni(NO_3_)_2_ in the non-normalized data, whereas no concentration-dependent differences were detected for the normalized values. Between the tested metal nitrates, significant differences were identified only between Cd(NO_3_)_2_ and Zn(NO_3_)_2_ for the non-normalized catalase activity and between Ni(NO_3_)_2_ and Zn((NO_3_)_2_ for the normalized catalase activity (Supplementary Tables S4 and S5).

For the non-normalized guaiacol peroxidase activity, Cd and Co exhibited consistently low activities at both tested concentrations. Cu, Ni, and Pb showed a decrease in activity with increasing concentration, whereas Cr and Zn displayed the opposite trend, with higher activity at the higher concentration. In contrast, Mn maintained a consistently elevated guaiacol peroxidase activity at both concentrations, showing no apparent concentration-dependent change. For the normalized data, Cd and Pb exhibited higher guaiacol peroxidase activity at the lower exposure concentration, whereas all other metals showed increased normalized activity with increasing concentration. No significant differences between the tested metal nitrates were observed for either the non-normalized or normalized data (Supplementary Tables S4 and S5). Between the two tested concentrations of the same metal nitrate, significant differences were detected only for Cd(NO_3_)_2_, Co(NO_3_)_2_, and Cu(NO_3_)_2_ in the normalized guaiacol peroxidase activity.

Heavy-metal toxicity induces excessive production of reactive oxygen species (ROS), which damage cellular macromolecules such as DNA, proteins, and lipids, collectively leading to oxidative stress. This redox imbalance may arise through several mechanisms, including the inhibition of antioxidant enzymes through interaction with essential metal-binding sites [70]. Catalase is a key component of the antioxidant defense system, alongside guaiacol peroxidase, and it plays a central role in detoxifying hydrogen peroxide (H_2_O_2_), a major reactive oxygen species generated under heavy-metal stress [71,72]. Their activity is often induced in heavy-metal exposure, reflecting activation of the plant’s enzymatic defense machinery under oxidative stress conditions [73,74].

The biochemical responses reflected compound-dependent phytotoxicity. Severe toxicity was associated with reduced chlorophyll, very low or undetectable protein levels, and weak antioxidant activity, whereas increased reducing sugar and catalase activity in some treatments suggest activation of stress-response mechanisms. Overall, the observed alterations in photosynthetic pigments and biochemical parameters indicate that exposure to the tested metal nitrates affected the physiological status of *L. minor*. These responses are consistent with possible disturbances in photosynthetic processes, primary metabolism, and antioxidant defenses, although the specific mechanisms underlying these effects were not directly investigated in the present study.

### 3.3. SSD-Based Species Sensitivity Comparison

**Figure 4:**
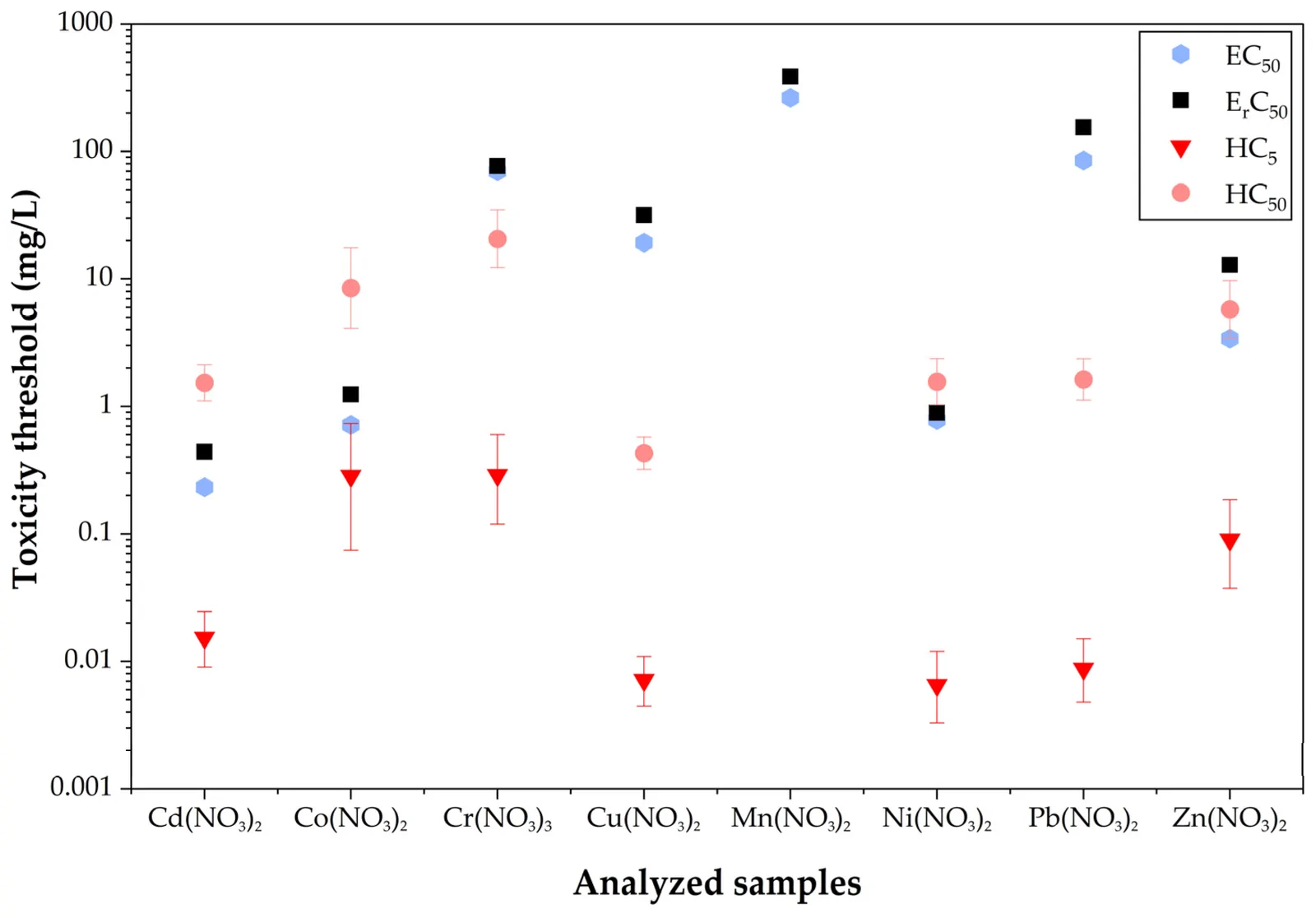
Toxicity threshold (mg/L) for each potentially toxic metal salt analyzed. EC_50_, together with hazardous concentration values for HC_5_ and HC_50_, are represented. Each bar represents the range defined by the lower and upper estimates for the hazardous concentration values, while the symbols represent the median value.

Species sensitivity distributions (SSDs) were constructed to estimate the hazardous concentrations (HC_5_ and HC_50_) for each metal nitrate. Figure 4 presents a comparison between the EC_50_ values determined in this study and the HC_5_ and HC_50_ values estimated using ETX software. With the exception of manganese, for which no suitable data was available in the ECOTOX database, all EC_50_ and E_r_C_50_ values exceeded the HC_5_. For Cd, Co, Ni, and Zn (EC_50_ only) values fell between the HC_5_ and HC_50_, indicating that *L. minor*, used as testing organism in this study, exhibits average sensitivity relative to the species represented in the SSD. In contrast, values for Cr, Cu, and Pb exceeded HC_50_, suggesting that *L. minor* is less sensitive to these metals than most species in the SSD. These results highlight that the sensitivity of *L. minor* varies depending on the metal, ranging from typical (mid-range) to relatively low sensitivity within the broader species sensitivity distribution.

To contextualize the present findings, relevant literature was reviewed. Articles describing the effects of heavy metals on *L. minor* were identified through Google Scholar using search terms such as “*Lemna minor* heavy metal toxicity.” For individual metals, the Web of Science Advanced Search was employed using the query builder with the format TI = (metal name*) AND ALL = (Lemna*) AND ALL = (EC50*). This approach was applied to a broad list of metals, including As, Bi, Cd, Co, Cr, Cu, Ga, Au, In, Ir, Fe, Pb, Hg, Mn, Mo, Ni, Os, Pd, Pt, Rh, Ru, Se, Ag, Te, Tl, Th, Sn, W, U, V, and Zn.

**Figure 5:**
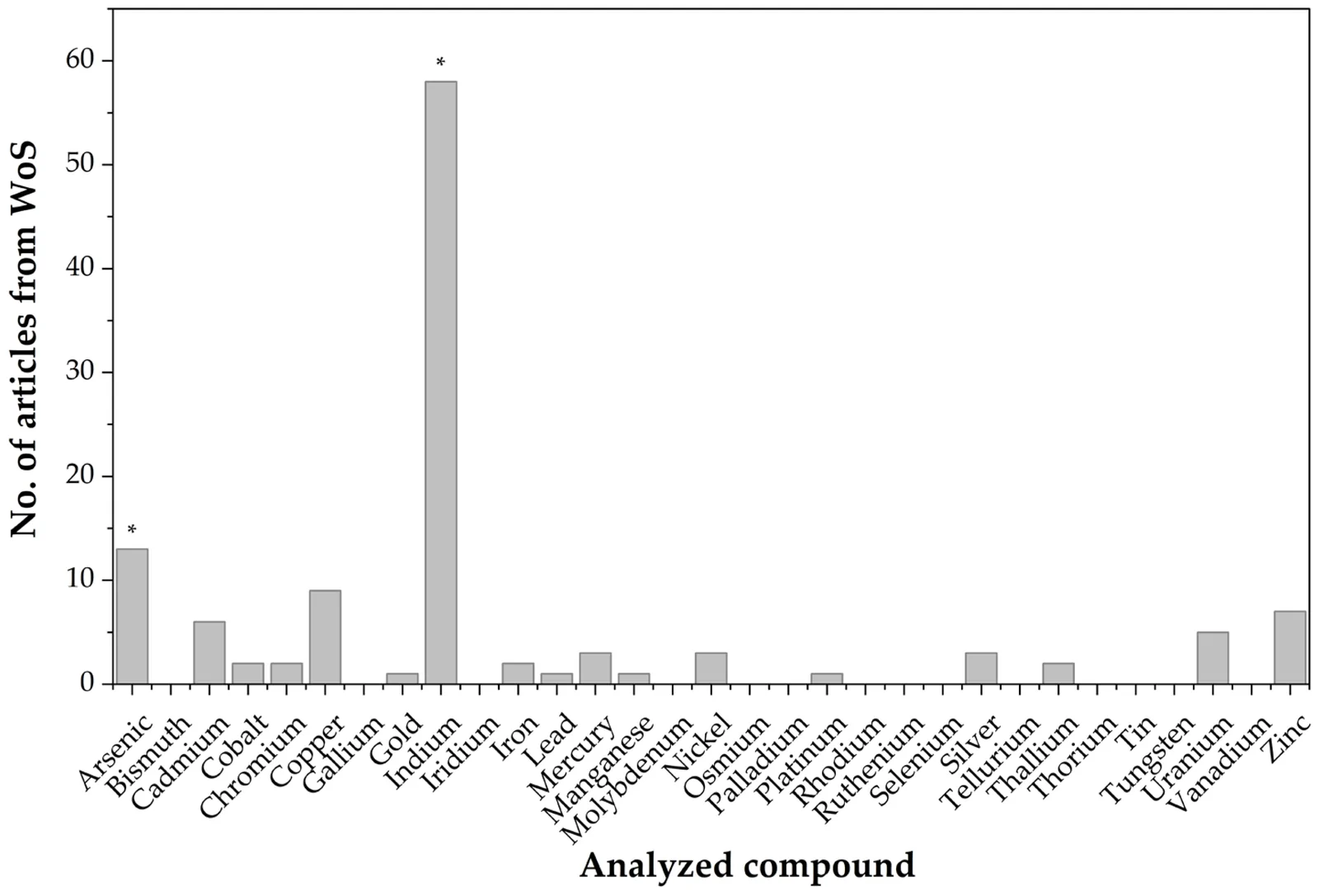
Number of published articles according to WoS Core Collection. The * symbol indicates number of studies for Arsenic and Indium, where the elemental notation also denotes the words “as” and “in”.

The data presented in Figure 5 indicates a relatively low number of studies reporting EC_50_ or E_r_C_50_ for *L. minor*, with fewer than ten publications per metal in most cases. Exceptions were observed for arsenic and indium, where elemental notation (“As” and “In”) led to additional articles being retrieved in the search. These findings suggest that relatively few studies have reported standardized EC_50_ or E_r_C_50_ values for *L. minor* for the investigated metals.

**Figure 6:**
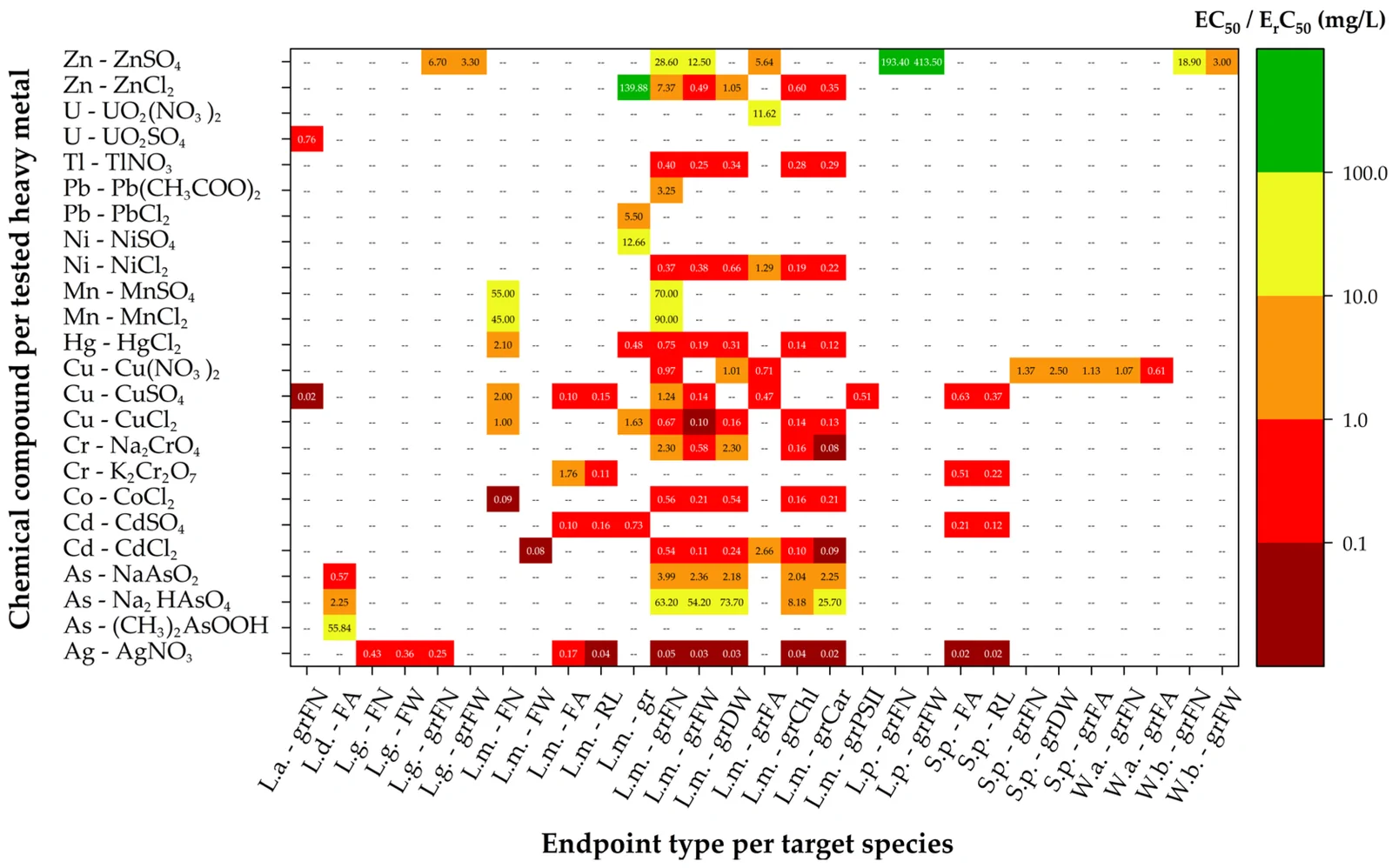
Heatmap of EC_50_/E_r_C_50_ values (mg/L) for different metals and chemical forms across *Lemna* species and endpoints. Columns represent species and endpoints, rows represent metals and chemical forms, and color indicates toxicological endpoint magnitude. The species are abbreviated as follows: L.a. = *Lemna aequinoctialis*; L.d. = *L. disperma*; L.g. = *L. gibba*; L.m. = *L. minor*; S.p. = *Spirodela polyrhiza*; W.a. = *Wolffia arrhiza*; W.b. = *W. brasiliensis*. The parameters used for the determination of the toxicological endpoints are abbreviated as FN = frond number; FW = fresh weight; DW = dry weight; FA = frond area; RL = root length; gr = growth rate; gr__ = growth rate calculated on the abbreviated parameter; Chl = chlorophyll content; Car = carotenoids content; and maximum quantum yield of photosystem II; double hyphens represent absence of data.

From each identified study [75,76,77,78,79,80,81,82,83,84,85,86,87,88,89,90,91,92,93], we extracted, where available, the EC_50_ or E_r_C_50_ values, along with the chemical form of the metal, the endpoint used to calculate the toxicological measure, and the *Lemna* species tested (Supplementary Table S1). These data were visualized as a heatmap (Figure 6), which illustrates that EC_50_ or E_r_C_50_ values can vary both between different chemical forms of the same metal, as observed for As and Zn, and between different endpoint types for the same chemical form, as seen for Ag, As, Cd, Cr, and Zn. These patterns highlight the importance of testing the same chemical form and using consistent endpoint calculations when comparing toxicity data. Consequently, although numerous studies report toxicological endpoints, direct comparisons among studies may be complicated by differences in the chemical forms and endpoints investigated.

**Figure 7:**
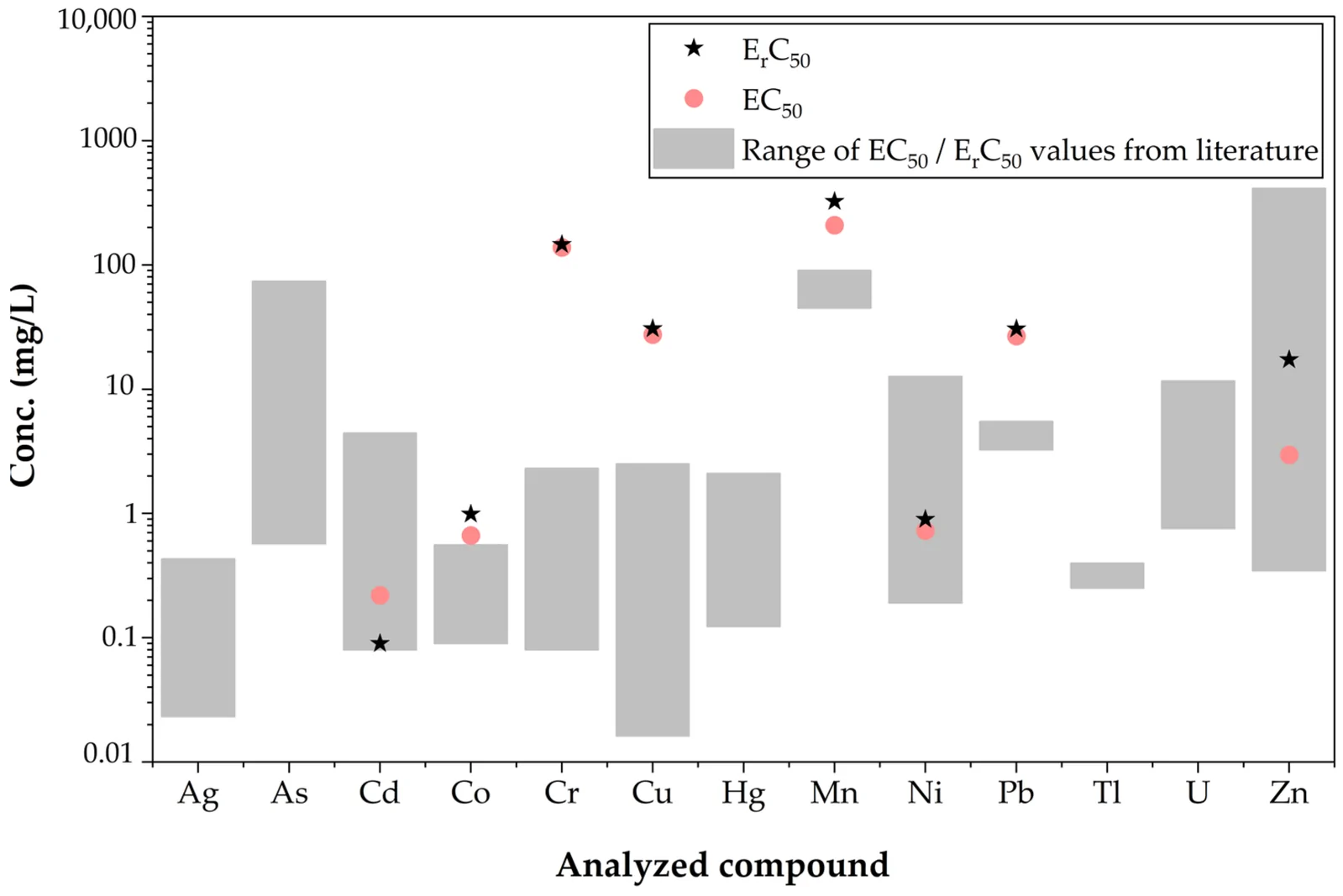
Comparison of toxicological endpoints from the present study and the range of such values from the literature.

A comparison of the experimental EC_50_ and E_r_C_50_ values obtained in this study with the ranges reported in the literature, considering all available chemical forms, is presented in Figure 7. Toxicological endpoints were reported in the literature for more than eight of the metals tested in the current study. For Cd, Ni, and Zn, the experimental values fall within the literature-reported ranges, while Co values were very similar. In contrast, the EC_50_ and E_r_C_50_ values for Cr, Cu, Mn, and Pb indicate lower toxicity in our experiments compared with previously published data. These differences may be attributable to variations in the chemical forms tested or to differences in the types of endpoints used to calculate toxicity.

Although phytotoxic effects were observed, *L. minor* is still able to accumulate heavy metals. However, the literature shows that both toxicity and accumulation are strongly influenced by environmental factors, particularly pH, which governs metal speciation, mobility, and bioavailability [94,95,96,97]. Such accumulation at the level of primary producers is ecologically relevant, as it represents a potential pathway for contaminant entry into aquatic food webs and subsequent transfer to higher trophic levels.

A limitation of the present study is that biochemical analyses were performed only at two exposure concentrations (0.001 and 1 mg/L). These concentrations were selected to represent environmentally relevant and elevated-exposure scenarios while providing sufficient plant biomass for reliable biochemical quantification. Consequently, the biochemical responses should be interpreted as representative of selected exposure levels rather than as complete concentration–response relationships. An additional limitation is that dissolved metal concentrations were not analytically verified during the exposure period. Therefore, the results are based on nominal concentrations, and potential changes in metal speciation, complexation with medium components, or precipitation during exposure cannot be excluded.

Future research should expand to include additional aquatic macrophyte species with different physiological and accumulation characteristics to improve the ecological relevance of toxicity assessments. In addition, integrating bioaccumulation and potential trophic transfer into multispecies experimental designs would allow a more realistic representation of contaminant movement across aquatic food webs, as pollutant transfer from *L. minor* to higher trophic levels has been previously demonstrated [98,99,100,101]. Beyond the effects of these heavy metals on *L. minor*, its low sensitivity, tolerance to pollutants, and high bioaccumulation capacity make this species a promising candidate for the bioremediation of wastewater, including heavy-metal-contaminated waters [94,102,103,104]. Accordingly, future studies should explore innovative bioremediation strategies using this plant, focusing on mechanistic aspects of metal uptake and tolerance under environmentally relevant conditions, as well as the management of metal-contaminated biomass generated after phytoremediation. The species is inexpensive, widely distributed, and fast-growing, making it a promising candidate for sustainable and safe solutions for both human health and the environment.

## 4. Conclusions

This study provides a comprehensive assessment of the ecotoxicity of eight metal nitrates in *L. minor* by integrating growth inhibition, biochemical responses, and species sensitivity analysis. Based on growth inhibition, the overall toxicity ranking of the investigated metal nitrates was Cd > Co > Ni > Zn > Cu > Cr > Pb > Mn according to EC_50_ values and Cd > Ni > Co > Zn > Cu > Cr > Pb > Mn according to E_r_C_50_ values, identifying Cd, Co, and Ni as the most phytotoxic compounds, whereas Mn exhibited the lowest toxicity. Furthermore, comparison of the experimentally determined EC_50_ values with reported environmental concentrations indicated that several of the investigated metals may occur at concentrations capable of causing phytotoxic effects in heavily contaminated aquatic environments.

The investigated metal nitrates also induced distinct compound-dependent biochemical responses, reflected by alterations in photosynthetic pigments, primary metabolites, and oxidative-stress-related enzymes, demonstrating that growth inhibition alone does not fully capture the complexity of plant responses to heavy-metal exposure. Furthermore, species sensitivity distribution analysis showed that the sensitivity of *L. minor* varied depending on the investigated metal, ranging from typical (mid-range) to relatively low sensitivity within the broader distribution of aquatic species.

Although heavy-metal toxicity in *L. minor* has been widely investigated, comparable studies integrating growth inhibition, biochemical biomarkers, and species sensitivity analysis under consistent exposure conditions remain limited. The present study contributes to filling this gap by providing an integrated dataset that facilitates a more comprehensive interpretation of the phytotoxicity of heavy-metal nitrates.

Overall, by integrating growth inhibition, biochemical responses, species sensitivity analysis, and comparison with reported environmental concentrations, this study provides a comprehensive evaluation of the phytotoxicity of metal nitrates and contributes to the ecological interpretation of laboratory toxicity data and their application in aquatic environmental risk assessment.

## Figures and Tables

**Table 1:** EC_50_ and E_r_C_50_ of tested metal nitrates.

Tested Sample	EC_50_ (mg/L)	E_r_C_50_ (mg/L)
Cd(NO_3_)_2_	0.23 ± 0.02 mg/L; CI: 0.19–0.29	0.44 ± 0.08 mg/L; CI: 0.30–0.70
Co(NO_3_)_2_	0.71 ± 0.15 mg/L; CI: 0.38–0.99	1.24 ± 0.43 mg/L; CI: 0.55–2.92
Cr(NO_3_)_2_	70.13 ± 17.05 mg/L; CI: 41.64–105.15	76.93 ± 8.55 mg/L; CI: 57.25–94.40
Cu(NO_3_)_2_	19.26 ± 3.90 mg/L; CI: 12.39–27.38	31.64 ± 3.42 mg/L; CI: 24.97–42.64
Mn(NO_3_)_2_	264.25 ± 54.39 mg/L; CI: 150.72–372.02	386.63 ± 49.73 mg/L; CI: 282.78–497.39
Ni(NO_3_)_2_	0.78 ± 0.03 mg/L; CI: 0.72–0.82	0.89 ± 0.04 mg/L; CI: 0.79–0.96
Pb(NO_3_)_2_	85.26 ± 48.13 mg/L; CI: 35.44–312.24	154.62 ± 56.74 mg/L; CI: 71.43–318.06
Zn(NO_3_)_2_	3.40 ± 0.33 mg/L; CI: 2.74–4.21	12.89 ± 3.33 mg/L; CI: 5.79–43.02

## Data Availability

The original contributions presented in the paper are included in the article and further inquiries can be directed to the corresponding author.

## Supplementary Materials

The following supporting information can be downloaded at https://www.mdpi.com/article/10.3390/toxics14090742/s1, Table S1: Literature data regarding EC_50_ values of toxic metals and metalloids for *Lemna* species. GR = growth rate; Figure S1: Dose–response curve of relative number of fronds (% of C−) and Cd(NO_3_)_2_ concentrations. Points labeled with different letters represent treatments that are significantly different from each other (*p* < 0.05; one-way ANOVA followed by post hoc test). Treatments sharing the same letter are not significantly different; Figure S2: Dose–response curve of relative number of fronds (% of C−) and Co(NO_3_)_2_ concentrations. Points labeled with different letters represent treatments that are significantly different from each other (*p* < 0.05; one-way ANOVA followed by post hoc test). Treatments sharing the same letter are not significantly different; Figure S3: Dose–response curve of relative number of fronds (% of C−) and Cr(NO_3_)_3_ concentrations. Points labeled with different letters represent treatments that are significantly different from each other (*p* < 0.05; one-way ANOVA followed by post hoc test). Treatments sharing the same letter are not significantly different; Figure S4: Dose–response curve of relative number of fronds (% of C−) and Cu(NO_3_)_2_ concentrations. Points labeled with different letters represent treatments that are significantly different from each other (*p* < 0.05; one-way ANOVA followed by post hoc test). Treatments sharing the same letter are not significantly different; Figure S5: Dose–response curve of relative number of fronds (% of C−) and Mn(NO_3_)_2_ concentrations. Points labeled with different letters represent treatments that are significantly different from each other (*p* < 0.05; one-way ANOVA followed by post hoc test). Treatments sharing the same letter are not significantly different; Figure S6: Dose–response curve of relative number of fronds (% of C−) and Ni(NO_3_)_2_ concentrations. Points labeled with different letters represent treatments that are significantly different from each other (*p* < 0.05; one-way ANOVA followed by post hoc test). Treatments sharing the same letter are not significantly different; Figure S7: Dose–response curve of relative number of fronds (% of C−) and Pb(NO_3_)_2_ concentrations. Points labeled with different letters represent treatments that are significantly different from each other (*p* < 0.05; one-way ANOVA followed by post hoc test). Treatments sharing the same letter are not significantly different; Figure S8: Dose–response curve of relative number of fronds (% of C−) and Zn(NO_3_)_2_ concentrations. Points labeled with different letters represent treatments that are significantly different from each other (*p* < 0.05; one-way ANOVA followed by post hoc test). Treatments sharing the same letter are not significantly different; Figure S9: Dose–response curve of average specific growth rates and Cd(NO_3_)_2_ concentrations. Points labeled with different letters represent treatments that are significantly different from each other (*p* < 0.05; one-way ANOVA followed by post hoc test). Treatments sharing the same letter are not significantly different; Figure S10: Dose–response curve of average specific growth rates and Co(NO_3_)_2_ concentrations. Points labeled with different letters represent treatments that are significantly different from each other (*p* < 0.05; one-way ANOVA followed by post hoc test). Treatments sharing the same letter are not significantly different; Figure S11: Dose–response curve of average specific growth rates and Cr(NO_3_)_3_ concentrations. Points labeled with different letters represent treatments that are significantly different from each other (*p* < 0.05; one-way ANOVA followed by post hoc test). Treatments sharing the same letter are not significantly different; Figure S12: Dose–response curve of average specific growth rates and Cu(NO_3_)_2_ concentrations. Points labeled with different letters represent treatments that are significantly different from each other (*p* < 0.05; one-way ANOVA followed by post hoc test). Treatments sharing the same letter are not significantly different; Figure S13: Dose–response curve of average specific growth rates and Mn(NO_3_)_2_ concentrations. Points labeled with different letters represent treatments that are significantly different from each other (*p* < 0.05; one-way ANOVA followed by post hoc test). Treatments sharing the same letter are not significantly different; Figure S14: Dose–response curve of average specific growth rates and Ni(NO_3_)_2_ concentrations. Points labeled with different letters represent treatments that are significantly different from each other (*p* < 0.05; one-way ANOVA followed by post hoc test). Treatments sharing the same letter are not significantly different; Figure S15: Dose–response curve of average specific growth rates and Pb(NO_3_)_2_ concentrations. Points labeled with different letters represent treatments that are significantly different from each other (*p* < 0.05; one-way ANOVA followed by post hoc test). Treatments sharing the same letter are not significantly different; Figure S16: Dose–response curve of average specific growth rates and Zn(NO_3_)_2_ concentrations. Points labeled with different letters represent treatments that are significantly different from each other (*p* < 0.05; one-way ANOVA followed by post hoc test). Treatments sharing the same letter are not significantly different; Table S2: Chlorophyll and carotenoid contents expressed as percentages of the negative control (mean ± SD), with compact letter displays (SCLDs) indicating statistical differences among treatments. LOD = limit of detection; Table S3: Chlorophyll and carotenoid contents normalized to fresh weight and expressed as percentages of the negative control (mean ± SD), with compact letter displays (SCLDs) indicating statistical differences among treatments. LOD = limit of detection; Table S4: Reducing sugar, proteins, catalase activity, and guaiacol peroxidase activity expressed as percentages of the negative control (mean ± SD), with compact letter displays (SCLDs) indicating statistical differences among treatments. LOD = limit of detection; Table S5: Reducing sugar, proteins, catalase activity, and guaiacol peroxidase activity normalized to fresh weight and expressed as percentages of the negative control (mean ± SD), with compact letter displays (SCLDs) indicating statistical differences among treatments. LOD = limit of detection.
