# Supplementary material for: Integrated Ecotoxicological Assessment of Metal Nitrates in Lemna minor Based on Growth Inhibition, Biochemical Responses, and Species Sensitivity Comparison
Source: Toxics. 2026 Aug 22;14(9):742. doi: 10.3390/toxics14090742 (PMC13611780; doi:10.3390/toxics14090742)
Supplement: Supplementary file 1 [file toxics-14-00742-s001.zip › toxics-4395913-supplementary.pdf]

toxics

Article

Integrated Ecotoxicological Assessment of Metal Nitrates in *Lemna minor* Based on Growth Inhibition, Biochemical Responses, and Species Sensitivity Comparison

Adina-Daniela Iachimov-Datcu [1,2], Diana-Maria Istrate [1], Lorena-Maria Toboșaru [1], Georgiana-Gabriela Doczi [1], Costel-Cristian Bulancea [1], Diana-Larisa Roman [1,2], Bianca-Vanesa Agachi [1,2,*] and Constantina-Bianca Vulpe [2,3]

[1] Department of Biology, Faculty of Chemistry, Biology, Geography, West University of Timisoara, Pestalozzi 16, 300115 Timisoara, Romania; daniela.iachimov@e-uvt.ro (A.-D.I.-D.); diana.istrate02@e-uvt.ro (D.-M.I.); lorena.tobosaru03@e-uvt.ro (L.-M.T.); doczigeorgiana10@gmail.com (G.-G.D.); bulancea.cristi2027@gmail.com (C.-C.B.); diana.roman@e-uvt.ro (D.-L.R.)
[2] One Health Initiative-Focused Multidisciplinary Biosciences Advanced Research Center, West University of Timisoara, Oituz 4, 300086 Timisoara, Romania; constantina.vulpe@e-uvt.ro
[3] Department of Scientific Research in Biology, Advanced Environmental Research Institute, West University of Timisoara, Oituz 4, 300086 Timisoara, Romania
* Correspondence: bianca.agachi@e-uvt.ro

Supplementary Materials

Table S1. Literature data regarding EC_{50} values of toxic metals and metalloids for duckweed species. GR = growth rate.

Element	Chemical Compound	EC_{50} (mg/L)	Endpoint Type	Species	Reference
Ag	$AgNO_3$	0.362	fresh weight	*Lemna gibba*	[1]
Ag	$AgNO_3$	0.430	frond number	*Lemna gibba*	[1]
Ag	$AgNO_3$	0.251	GR from frond number	*Lemna gibba*	[1]
Ag	$AgNO_3$	0.166	frond area	*Lemna minor*	[2]
Ag	$AgNO_3$	0.041	root length	*Lemna minor*	[2]
Ag	$AgNO_3$	0.023	frond area	*Spirodela polyrhiza*	[2]
Ag	$AgNO_3$	0.023	root length	*Spirodela polyrhiza*	[2]
Ag	$AgNO_3$	0.030	GR from frond number	*Lemna minor*	[3]
Ag	$AgNO_3$	0.037	GR from frond number	*Lemna minor*	[3]
Ag	$AgNO_3$	0.024	GR from carotenoid content	*Lemna minor*	[4]
Ag	$AgNO_3$	0.037	GR from chlorophyll content	*Lemna minor*	[4]
Ag	$AgNO_3$	0.031	GR from dry weight	*Lemna minor*	[4]
Ag	$AgNO_3$	0.030	GR from fresh weight	*Lemna minor*	[4]
Ag	$AgNO_3$	0.081	GR from frond number	*Lemna minor*	[4]
As	$(CH_3)_2AsOOH$	55.840	frond area	*Lemna disperma*	[5]
As	Na_2HAsO_4	2.250	frond area	*Lemna disperma*	[5]
As	Na_2HAsO_4	25.700	GR from carotenoid content	*Lemna minor*	[4]
As	Na_2HAsO_4	8.180	GR from chlorophyll content	*Lemna minor*	[4]
As	Na_2HAsO_4	73.700	GR from dry weight	*Lemna minor*	[4]
As	Na_2HAsO_4	54.200	GR from fresh weight	*Lemna minor*	[4]
As	Na_2HAsO_4	63.200	GR from frond number	*Lemna minor*	[4]
As	$NaAsO_2$	0.570	frond area	*Lemna disperma*	[5]
As	$NaAsO_2$	2.250	GR from carotenoid content	*Lemna minor*	[4]
As	$NaAsO_2$	2.040	GR from chlorophyll content	*Lemna minor*	[4]
As	$NaAsO_2$	2.180	GR from dry weight	*Lemna minor*	[4]

As	$NaAsO_2$	2.360	GR from fresh weight	*Lemna minor*	[4]
As	$NaAsO_2$	3.990	GR from frond number	*Lemna minor*	[4]
Cd	$CdCl_2$	4.418	GR from frond area	*Lemna minor*	[6]
Cd	$CdCl_2$	0.910	GR from frond area	*Lemna minor*	[7]
Cd	$CdCl_2$	0.080	fresh biomass	*Lemna minor*	[8]
Cd	$CdCl_2$	0.390	GR from frond number	*Lemna minor*	[8]
Cd	$CdCl_2$	0.092	GR from carotenoid content	*Lemna minor*	[4]
Cd	$CdCl_2$	0.102	GR from chlorophyll content	*Lemna minor*	[4]
Cd	$CdCl_2$	0.241	GR rate from dry weight	*Lemna minor*	[4]
Cd	$CdCl_2$	0.110	GR from fresh weight	*Lemna minor*	[4]
Cd	$CdCl_2$	0.323	GR from frond number	*Lemna minor*	[4]
Cd	$CdCl_2$	0.917	GR from frond number	*Lemna minor*	[9]
Cd	$CdSO_4$	0.100	frond area	*Lemna minor*	[2]
Cd	$CdSO_4$	0.155	root length	*Lemna minor*	[2]
Cd	$CdSO_4$	0.205	frond area	*Spirodela polyrhiza*	[2]
Cd	$CdSO_4$	0.122	root length	*Spirodela polyrhiza*	[2]
Cd	$CdSO_4$	0.730	GR	*Lemna minor*	[10]
Co	$CoCl_2$	0.090	frond number	*Lemna minor*	[11]
Co	$CoCl_2$	0.205	GR from carotenoid content	*Lemna minor*	[4]
Co	$CoCl_2$	0.163	GR from chlorophyll content	*Lemna minor*	[4]
Co	$CoCl_2$	0.542	GR from dry weight	*Lemna minor*	[4]
Co	$CoCl_2$	0.212	GR from fresh weight	*Lemna minor*	[4]
Co	$CoCl_2$	0.557	GR from frond number	*Lemna minor*	[4]
Cr	$K_2Cr_2O_7$	1.756	frond area	*Lemna minor*	[2]
Cr	$K_2Cr_2O_7$	0.109	root length	*Lemna minor*	[2]
Cr	$K_2Cr_2O_7$	0.507	frond area	*Spirodela polyrhiza*	[2]
Cr	$K_2Cr_2O_7$	0.219	root length	*Spirodela polyrhiza*	[2]
Cr	Na_2CrO_4	0.080	GR from carotenoid content	*Lemna minor*	[4]
Cr	Na_2CrO_4	0.155	GR from chlorophyll content	*Lemna minor*	[4]
Cr	Na_2CrO_4	2.300	GR from dry weight	*Lemna minor*	[4]
Cr	Na_2CrO_4	0.584	GR from fresh weight	*Lemna minor*	[4]
Cr	Na_2CrO_4	2.300	GR from frond number	*Lemna minor*	[4]
Cu	$Cu(NO_3)_2$	1.010	GR from dry weight	*Lemna minor*	[12]
Cu	$Cu(NO_3)_2$	0.710	GR from frond area	*Lemna minor*	[12]
Cu	$Cu(NO_3)_2$	0.970	GR from frond number	*Lemna minor*	[12]
Cu	$Cu(NO_3)_2$	2.500	GR from dry weight	*Spirodela polyrhiza*	[12]
Cu	$Cu(NO_3)_2$	1.130	GR from frond area	*Spirodela polyrhiza*	[12]
Cu	$Cu(NO_3)_2$	1.370	GR from frond number	*Spirodela polyrhiza*	[12]
Cu	$Cu(NO_3)_2$	0.610	GR from frond area	*Wolffia arrhiza*	[12]
Cu	$Cu(NO_3)_2$	1.070	GR from frond number	*Wolffia arrhiza*	[12]
Cu	$CuCl_2$	1.627	GR	*Lemna minor*	[10]
Cu	$CuCl_2$	0.132	GR from carotenoid content	*Lemna minor*	[4]
Cu	$CuCl_2$	0.136	GR from chlorophyll content	*Lemna minor*	[4]
Cu	$CuCl_2$	0.157	GR from dry weight	*Lemna minor*	[4]
Cu	$CuCl_2$	0.095	GR from fresh weight	*Lemna minor*	[4]
Cu	$CuCl_2$	0.330	GR from frond number	*Lemna minor*	[4]
Cu	$CuCl_2$	1.000	frond number	*Lemna minor*	[13]
Cu	$CuCl_2$	1.000	GR from frond number	*Lemna minor*	[13]
Cu	$CuSO_4$	0.470	GR from frond area	*Lemna minor*	[7]
Cu	$CuSO_4$	0.100	frond area	*Lemna minor*	[2]
Cu	$CuSO_4$	0.145	root length	*Lemna minor*	[2]
Cu	$CuSO_4$	0.630	frond area	*Spirodela polyrhiza*	[2]
Cu	$CuSO_4$	0.365	root length	*Spirodela polyrhiza*	[2]

https://doi.org/10.3390/toxics14090742

Cu	$CuSO_4$	0.016	GR from frond number	*Lemna aequinoctialis*	[14]
Cu	$CuSO_4$	0.144	GR from fresh weight	*Lemna minor*	[15]
Cu	$CuSO_4$	0.145	GR from frond number	*Lemna minor*	[15]
Cu	$CuSO_4$	0.513	GR from maximum quantum yield of photosystem II	*Lemna minor*	[15]
Cu	$CuSO_4$	1.566	GR from frond number	*Lemna minor*	[9]
Cu	$CuSO_4$	2.000	frond number	*Lemna minor*	[13]
Cu	$CuSO_4$	2.000	GR from frond number	*Lemna minor*	[13]
Hg	$HgCl_2$	0.390	GR from dry weight	*Lemna minor*	[16]
Hg	$HgCl_2$	0.810	GR from frond number	*Lemna minor*	[16]
Hg	$HgCl_2$	2.100	frond number	*Lemna minor*	[17]
Hg	$HgCl_2$	0.480	GR	*Lemna minor*	[18]
Hg	$HgCl_2$	0.123	GR from carotenoid content	*Lemna minor*	[4]
Hg	$HgCl_2$	0.135	GR from chlorophyll content	*Lemna minor*	[4]
Hg	$HgCl_2$	0.221	GR from dry weight	*Lemna minor*	[4]
Hg	$HgCl_2$	0.191	GR from fresh weight	*Lemna minor*	[4]
Hg	$HgCl_2$	0.683	GR from frond number	*Lemna minor*	[4]
Mn	$MnCl_2$	45.000	frond number	*Lemna minor*	[13]
Mn	$MnCl_2$	90.000	GR from frond number	*Lemna minor*	[13]
Mn	$MnSO_4$	55.000	frond number	*Lemna minor*	[13]
Mn	$MnSO_4$	70.000	GR from frond number	*Lemna minor*	[13]
Ni	$NiCl_2$	1.290	GR from frond area	*Lemna minor*	[7]
Ni	$NiCl_2$	0.218	GR from carotenoid content	*Lemna minor*	[4]
Ni	$NiCl_2$	0.191	GR from chlorophyll content	*Lemna minor*	[4]
Ni	$NiCl_2$	0.655	GR from dry weight	*Lemna minor*	[4]
Ni	$NiCl_2$	0.384	GR from fresh weight	*Lemna minor*	[4]
Ni	$NiCl_2$	0.370	GR from frond number	*Lemna minor*	[4]
Ni	$NiSO_4$	12.659	growth rate	*Lemna minor*	[10]
Pb	$Pb(CH_3COO)_2$	3.253	GR from frond number	*Lemna minor*	[9]
Pb	$PbCl_2$	5.500	GR	*Lemna minor*	[18]
Tl	$TlNO_3$	0.288	GR from carotenoid content	*Lemna minor*	[4]
Tl	$TlNO_3$	0.277	GR from chlorophyll content	*Lemna minor*	[4]
Tl	$TlNO_3$	0.338	GR from dry weight	*Lemna minor*	[4]
Tl	$TlNO_3$	0.251	GR from fresh weight	*Lemna minor*	[4]
Tl	$TlNO_3$	0.397	GR from frond number	*Lemna minor*	[4]
U	$UO_2(NO_3)_2$	11.624	GR from frond area	*Lemna minor*	[6]
U	UO_2SO_4	0.758	GR from frond number	*Lemna aequinoctialis*	[14]
Zn	$ZnCl_2$	139.884	GR	*Lemna minor*	[10]
Zn	$ZnCl_2$	0.347	GR from carotenoid content	*Lemna minor*	[4]
Zn	$ZnCl_2$	0.601	GR from chlorophyll content	*Lemna minor*	[4]
Zn	$ZnCl_2$	1.050	GR from dry weight	*Lemna minor*	[4]
Zn	$ZnCl_2$	0.486	GR from fresh weight	*Lemna minor*	[4]
Zn	$ZnCl_2$	0.909	GR from frond number	*Lemna minor*	[4]
Zn	$ZnCl_2$	13.829	GR from frond number	*Lemna minor*	[9]
Zn	$ZnSO_4$	5.640	GR from frond area	*Lemna minor*	[7]
Zn	$ZnSO_4$	3.300	GR from fresh weight	*Lemna gibba*	[19]
Zn	$ZnSO_4$	6.700	GR from frond number	*Lemna gibba*	[19]
Zn	$ZnSO_4$	12.500	GR from fresh weight	*Lemna minor*	[19]
Zn	$ZnSO_4$	28.600	GR from frond number	*Lemna minor*	[19]
Zn	$ZnSO_4$	413.500	GR fresh weight	*Lemna punctata*	[19]
Zn	$ZnSO_4$	193.400	GR from frond number	*Lemna punctata*	[19]
Zn	$ZnSO_4$	3.000	GR fresh weight	*Wolfflia brasiliensis*	[19]
Zn	$ZnSO_4$	18.900	GR rate from frond number	*Wolfflia brasiliensis*	[19]

https://doi.org/10.3390/toxics14090742

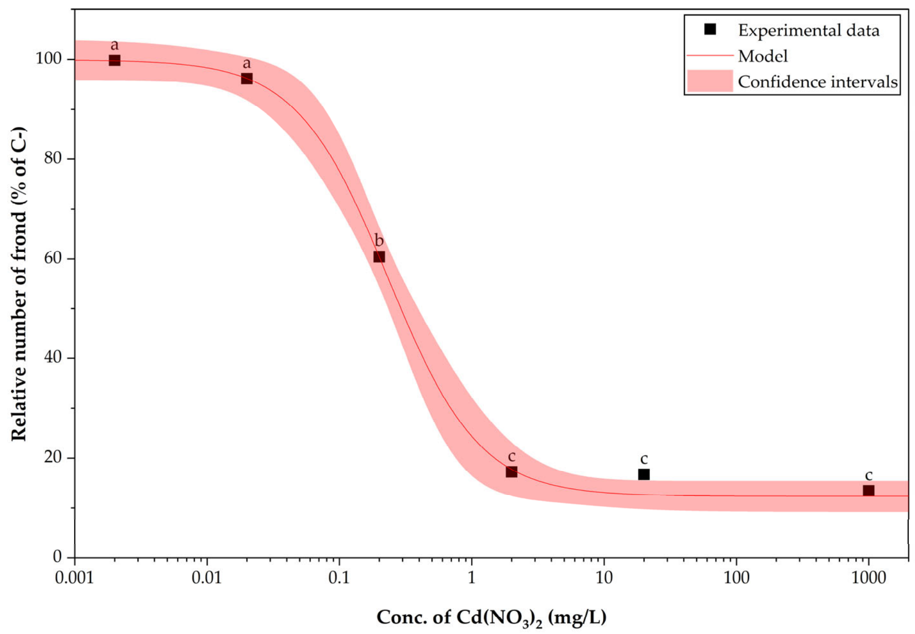

Figure S1. Dose-response curve of relative number of fronds (% of C-) and Cd(NO3)2 concentrations. Points labeled with different letters represent treatments that are significantly different from each other ($p < 0.05$; one- way ANOVA followed by post hoc test). Treatments sharing the same letter are not significantly different.

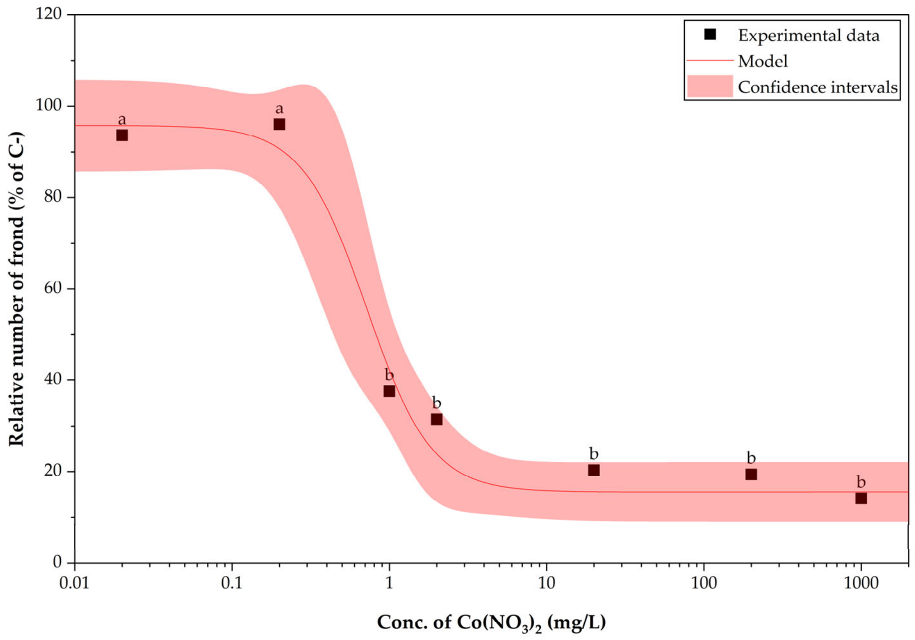

Figure S2. Dose-response curve of relative number of fronds (% of C-) and Co(NO3)2 concentrations. Points labeled with different letters represent treatments that are significantly different from each other ($p < 0.05$; one- way ANOVA followed by post hoc test). Treatments sharing the same letter are not significantly different.

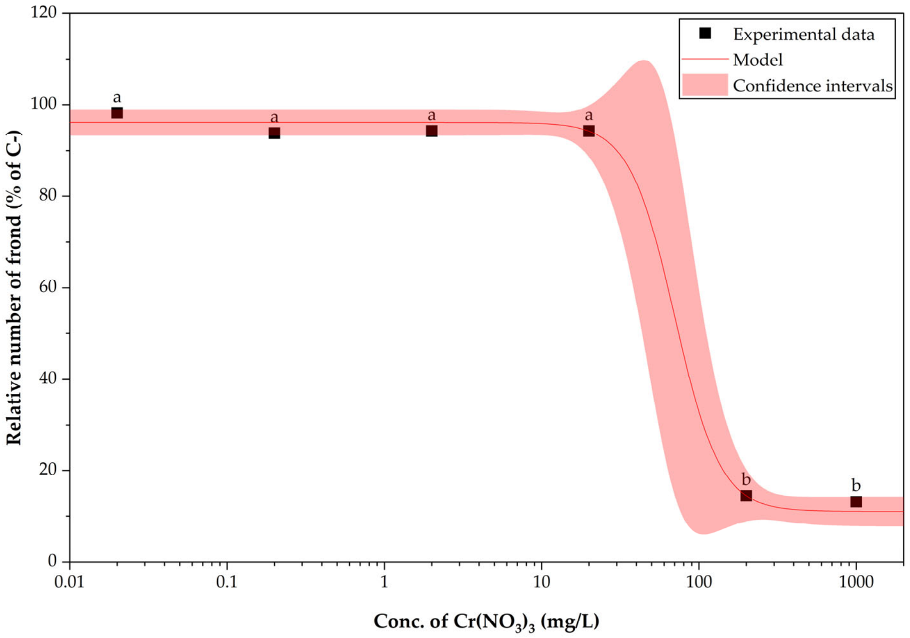

Figure S3. Dose-response curve of relative number of fronds (% of C-) and $Cr(NO_3)_3$ concentrations. Points labeled with different letters represent treatments that are significantly different from each other ($p < 0.05$; one- way ANOVA followed by post hoc test). Treatments sharing the same letter are not significantly different.

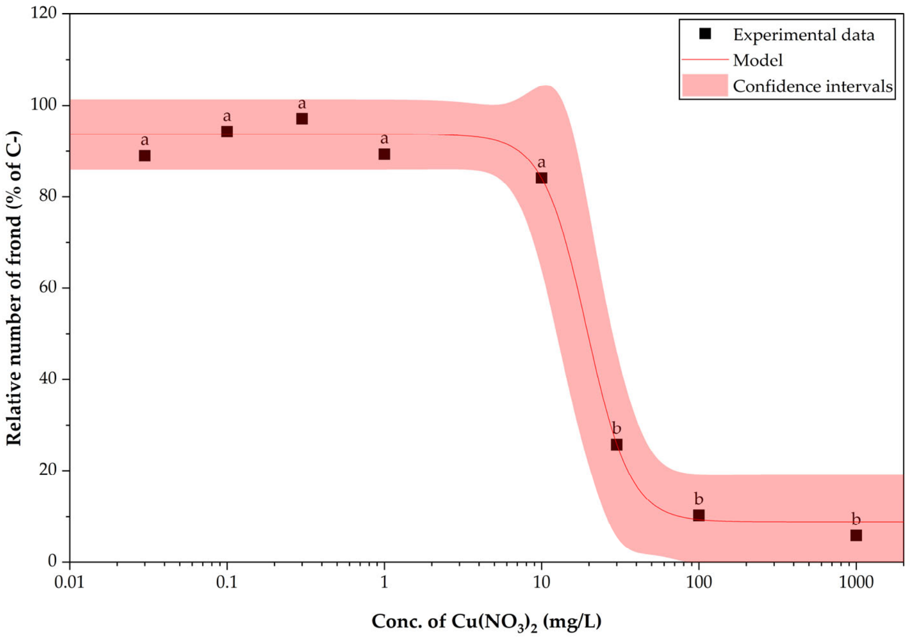

Figure S4. Dose-response curve of relative number of fronds (% of C-) and $Cu(NO_3)_2$ concentrations. Points labeled with different letters represent treatments that are significantly different from each other ($p < 0.05$; one- way ANOVA followed by post hoc test). Treatments sharing the same letter are not significantly different.

https://doi.org/10.3390/toxics14090742

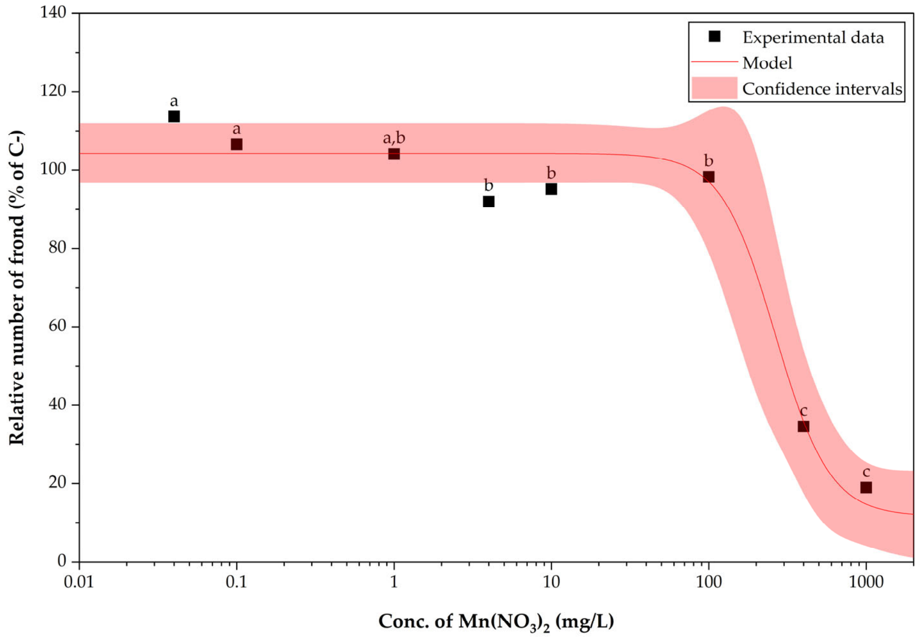

Figure S5. Dose-response curve of relative number of fronds (% of C-) and $Mn(NO_3)_2$ concentrations. Points labeled with different letters represent treatments that are significantly different from each other ($p < 0.05$; one- way ANOVA followed by post hoc test). Treatments sharing the same letter are not significantly different.

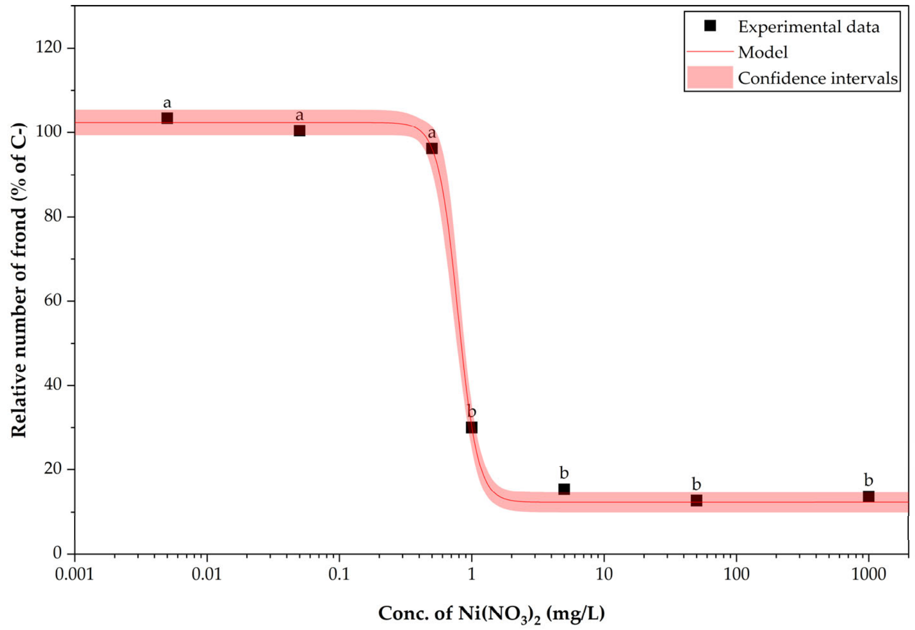

Figure S6. Dose-response curve of relative number of fronds (% of C-) and $Ni(NO_3)_2$ concentrations. Points labeled with different letters represent treatments that are significantly different from each other ($p < 0.05$; one- way ANOVA followed by post hoc test). Treatments sharing the same letter are not significantly different.

https://doi.org/10.3390/toxics14090742

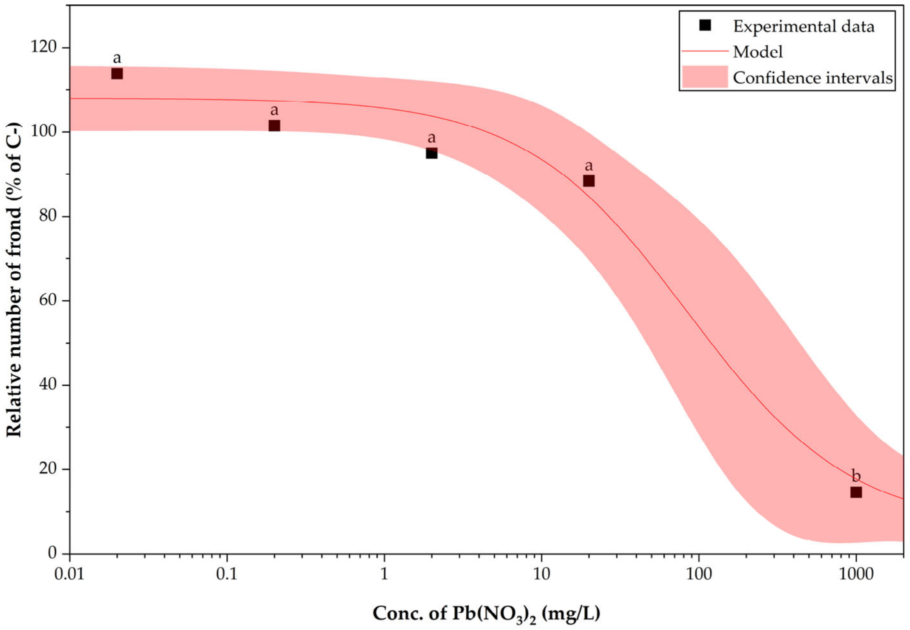

Figure S7. Dose-response curve of relative number of fronds (% of C-) and $Pb(NO_3)_2$ concentrations. Points labeled with different letters represent treatments that are significantly different from each other ($p < 0.05$; one- way ANOVA followed by post hoc test). Treatments sharing the same letter are not significantly different.

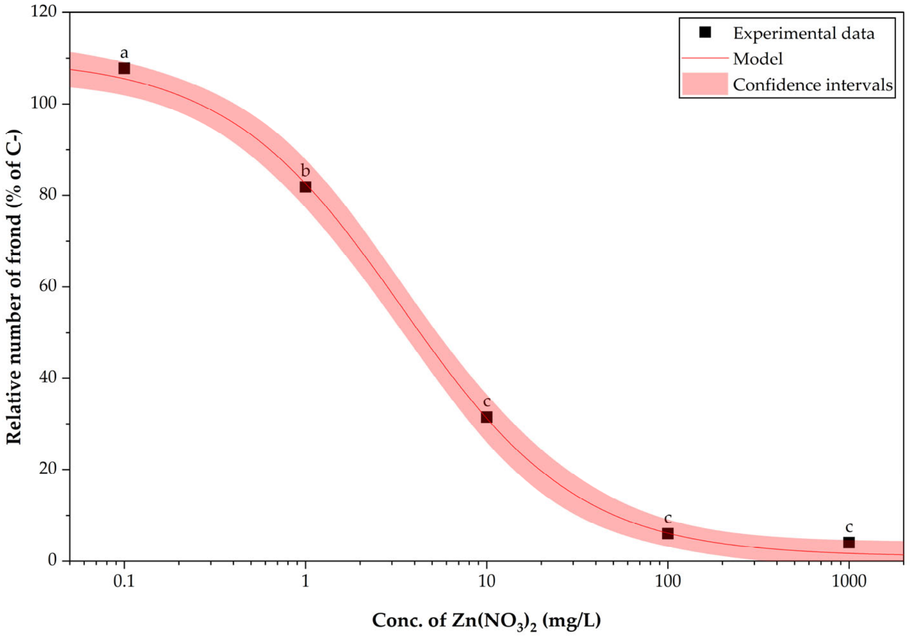

Figure S8. Dose-response curve of relative number of fronds (% of C-) and $Zn(NO_3)_2$ concentrations. Points labeled with different letters represent treatments that are significantly different from each other ($p < 0.05$; one- way ANOVA followed by post hoc test). Treatments sharing the same letter are not significantly different.

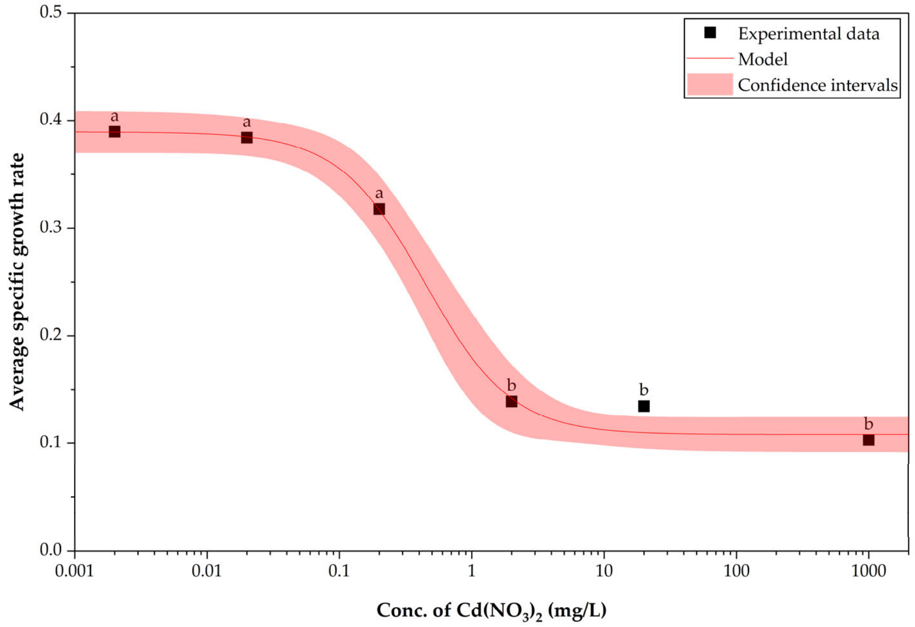

Figure S9. Dose-response curve of average specific growth rates and $Cd(NO_3)_2$ concentrations. Points labeled with different letters represent treatments that are significantly different from each other ($p < 0.05$; one- way ANOVA followed by post hoc test). Treatments sharing the same letter are not significantly different.

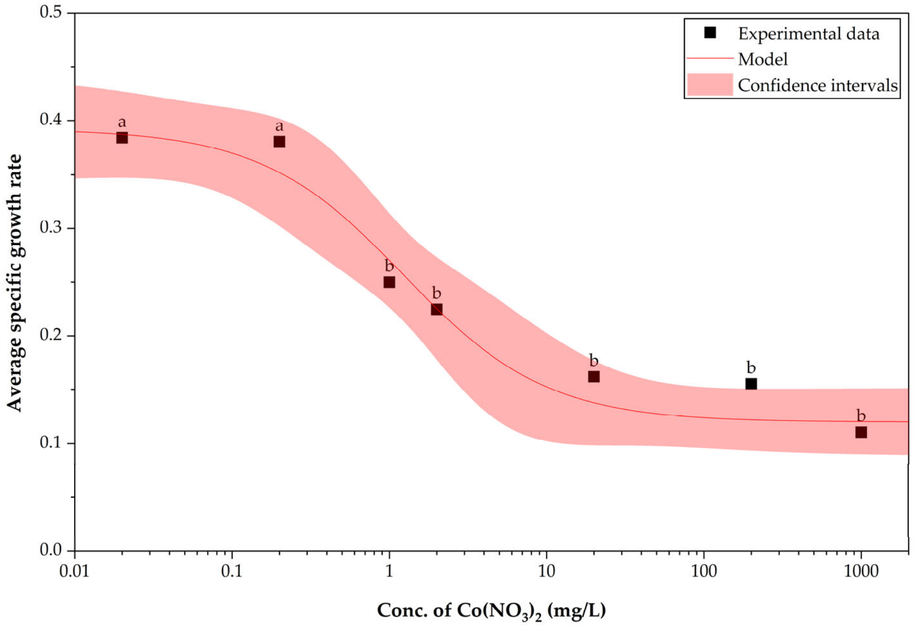

Figure S10. Dose-response curve of average specific growth rates and $Co(NO_3)_2$ concentrations. Points labeled with different letters represent treatments that are significantly different from each other ($p < 0.05$; one- way ANOVA followed by post hoc test). Treatments sharing the same letter are not significantly different.

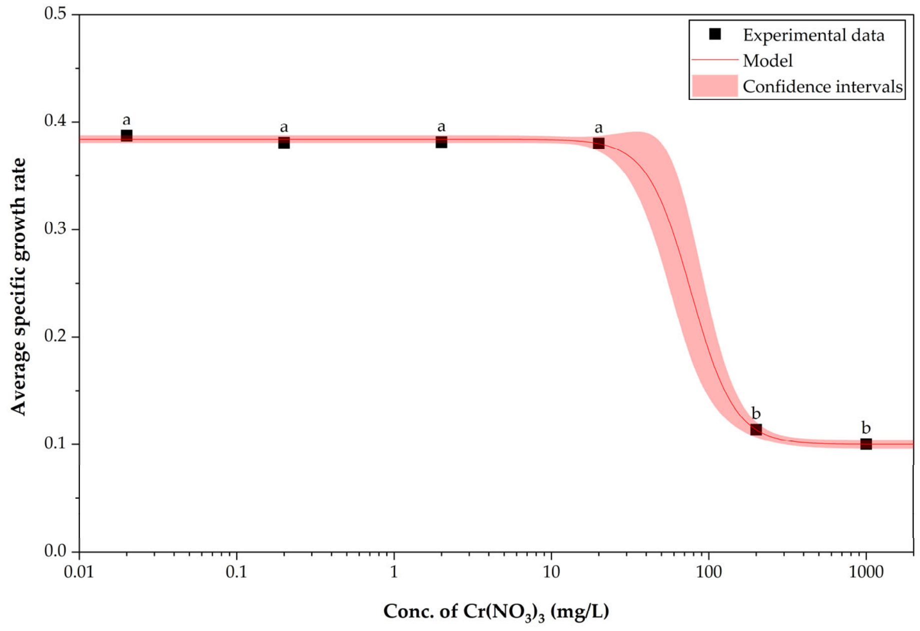

Figure S11. Dose-response curve of average specific growth rates and $Cr(NO_3)_3$ concentrations. Points labeled with different letters represent treatments that are significantly different from each other ($p < 0.05$; one- way ANOVA followed by post hoc test). Treatments sharing the same letter are not significantly different.

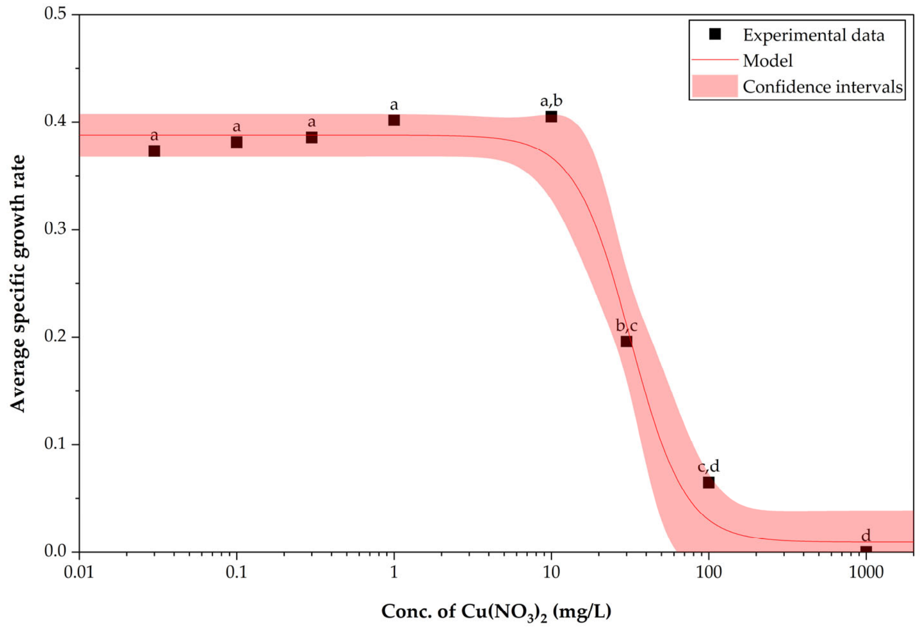

Figure S12. Dose-response curve of average specific growth rates and $Cu(NO_3)_2$ concentrations. Points labeled with different letters represent treatments that are significantly different from each other ($p < 0.05$; one- way ANOVA followed by post hoc test). Treatments sharing the same letter are not significantly different.

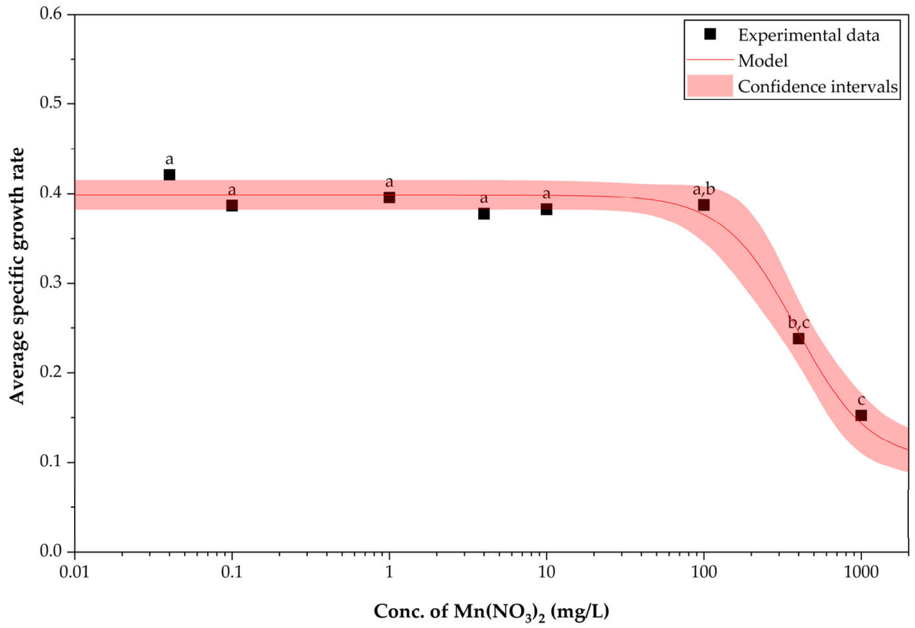

Figure S13. Dose-response curve of average specific growth rates and $Mn(NO_3)_2$ concentrations. Points labeled with different letters represent treatments that are significantly different from each other ($p < 0.05$; one- way ANOVA followed by post hoc test). Treatments sharing the same letter are not significantly different.

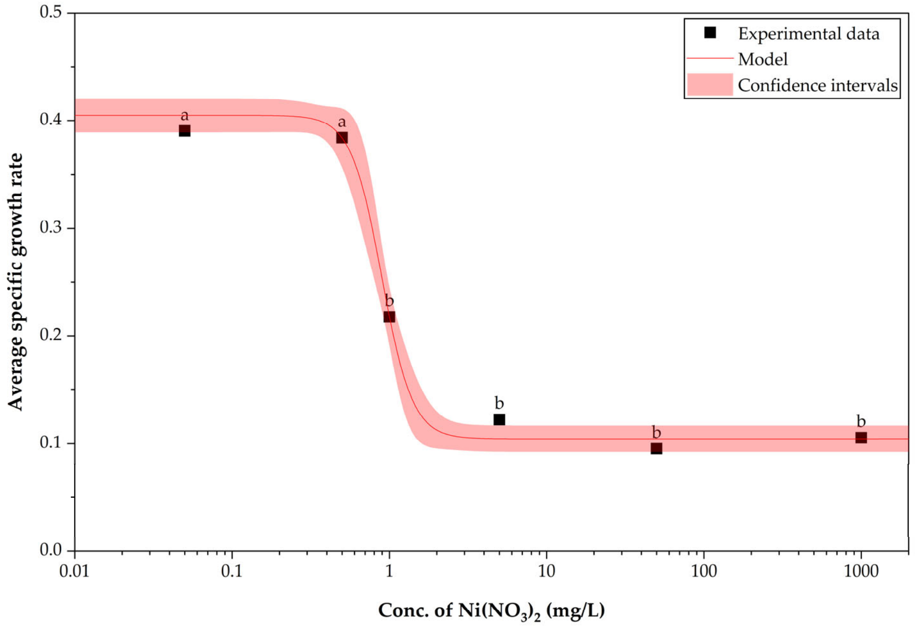

Figure S14. Dose-response curve of average specific growth rates and $Ni(NO_3)_2$ concentrations. Points labeled with different letters represent treatments that are significantly different from each other ($p < 0.05$; one- way ANOVA followed by post hoc test). Treatments sharing the same letter are not significantly different.

https://doi.org/10.3390/toxics14090742

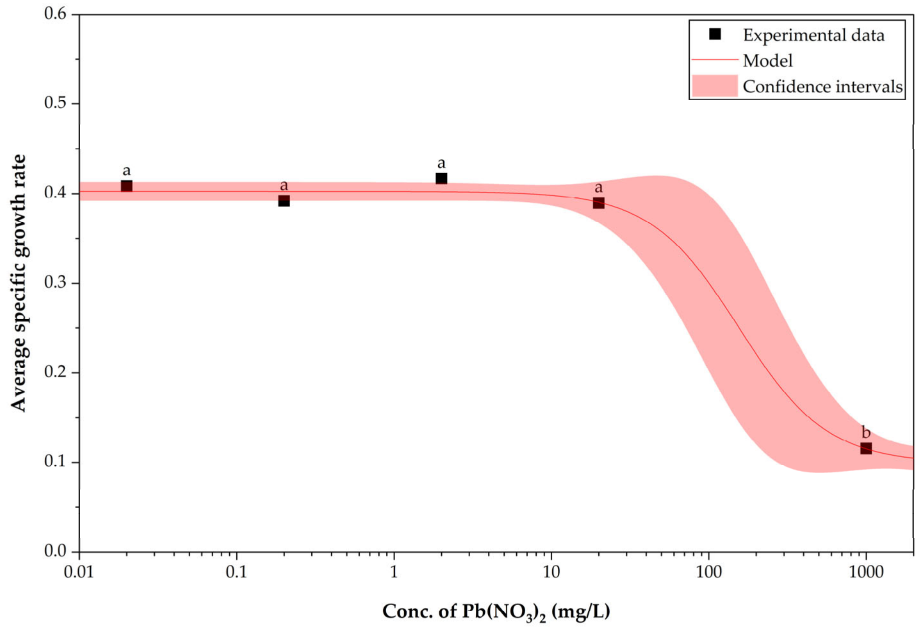

Figure S15. Dose-response curve of average specific growth rates and Pb(NO3)2 concentrations. Points labeled with different letters represent treatments that are significantly different from each other ($p < 0.05$; one- way ANOVA followed by post hoc test). Treatments sharing the same letter are not significantly different.

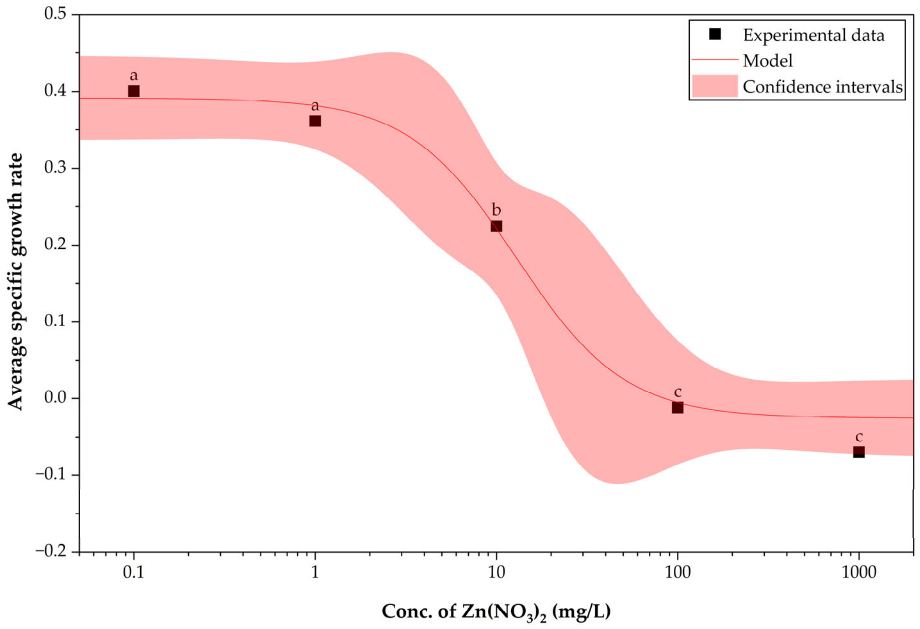

Figure S16. Dose-response curve of average specific growth rates and Zn(NO3)2 concentrations. Points labeled with different letters represent treatments that are significantly different from each other ($p < 0.05$; one- way ANOVA followed by post hoc test). Treatments sharing the same letter are not significantly different.

Table S2. Chlorophyll and carotenoid contents expressed as percentages of the negative control (mean ± SD), with compact letter displays (SCLD) indicating statistical differences among treatments. LOD = limit of detection

Tested sample	Chl a % C-	Chl a SCLD	Chl b % C-	Chl b SCLD	Chl a+b % C-	Chl a+b SCLD	Car x+c % C-	Car x+c SCLD
Negative control	100.00 ± 0.87%	abcde	100.00 ± 1.65%	abcdef	100.00 ± 1.06%	abcd	100.00 ± 0.72%	ab
$Cd(NO_3)_2$ 0,001 mg/L	92.31 ± 10.83%	abcde	88.97 ± 6.97%	abcdef	86.94 ± 6.72%	abcd	59.47 ± 5.78%	abcd
$Cd(NO_3)_2$ 1 mg/L	6.62 ± 0.21%	abc	23.64 ± 0.73%	abd	11.95 ± 0.31%	ab	< LOD	-
$Co(NO_3)_2$ 0,001 mg/L	118.55 ± 7.47%	de	118.64 ± 12.03%	acef	118.58 ± 8.62%	cd	81.20 ± 4.96%	abcd
$Co(NO_3)_2$ 1 mg/L	116.46 ± 7.21%	ade	121.37 ± 6.78%	cef	118.00 ± 7.02%	cd	86.08 ± 5.99%	abc
$Cr(NO_3)_3$ 0,001 mg/L	6.42 ± 0.13%	bc	23.94 ± 1.52%	abcd	11.91 ± 0.46%	ab	< LOD	-
$Cr(NO_3)_3$ 1 mg/L	123.78 ± 6.50%	de	123.87 ± 7.70%	ef	123.81 ± 6.75%	cd	88.19 ± 4.97%	ab
$Cu(NO_3)_2$ 0.001 mg/L	55.42 ± 4.05%	abcde	62.36 ± 3.72%	abcdef	57.60 ± 3.94%	abcd	35.37 ± 2.94%	acd
$Cu(NO_3)_2$ 1 mg/L	5.86 ± 0.13%	b	21.97 ± 0.45%	b	10.91 ± 0.21%	a	< LOD	-
$Mn(NO_3)_2$ 0.001 mg/L	106.98 ± 8.41%	acde	104.58 ± 6.95%	acdef	106.23 ± 7.94%	bcd	73.34 ± 6.32%	abcd
$Mn(NO_3)_2$ 1 mg/L	125.77 ± 8.52%	de	133.16 ± 13.07%	cef	128.29 ± 10.29%	cd	99.56 ± 24.08%	ab
$Ni(NO_3)_2$ 0,001 mg/L	147.10 ± 11.95%	d	151.94 ± 11.46%	e	148.62 ± 11.80%	c	131.05 ± 11.27%	b
$Ni(NO_3)_2$ 1 mg/L	10.56 ± 0.45%	abcde	25.93 ± 0.41%	abcdef	15.37 ± 0.42%	abcd	3.79 ± 0.59%	cd
$Pb(NO_3)_2$ 0.001 mg/L	7.47 ± 0.24%	abce	23.80 ± 0.42%	abcdf	12.59 ± 0.24%	abd	< LOD	-
$Pb(NO_3)_2$ 1 mg/L	93.55 ± 6.66%	abcde	98.23 ± 5.86%	abcdef	95.02 ± 6.39%	abcd	68.14 ± 5.40%	abcd
$Zn(NO_3)_2$ 0.001 mg/L	6.29 ± 0.70%	bc	23.21 ± 1.82%	bd	11.05 ± 0.55%	ab	< LOD	
$Zn(NO_3)_2$ 1 mg/L	9.01 ± 0.47%	abcde	27.23 ± 1.99%	abcdef	14.72 ± 0.82%	abcd	2.93 ± 0.46%	d
Positive control	7.73 ± 0.78%	abce	23.85 ± 0.32%	abcdef	12.39 ± 0.34%	abcd	< LOD	-

Table S3. Chlorophyll and carotenoid contents normalized to fresh weight and expressed as percentages of the negative control (mean ± SD), with compact letter displays (SCLD) indicating statistical differences among treatments. LOD = limit of detection

Tested sample	Chl a % C-	Chl a SCLD	Chl b % C-	Chl b SCLD	Chl a+b % C-	Chl a+b SCLD	Car x+c % C-	Car x+c SCLD
Negative control	100.00 ± 0.87%	abcd	100.00 ± 1.65%	a	100.00 ± 1.06%	ab	100.00 ± 0.72%	a
$Cd(NO_3)_2$ 0,001 mg/L	172.32 ± 20.22%	a	166.09 ± 13.02%	a	162.31 ± 12.55%	ab	107.66 ± 22.38%	a
$Cd(NO_3)_2$ 1 mg/L	17.99 ± 0.57%	abcdef	64.23 ± 1.97%	a	32.48 ± 0.84%	abcd	< LOD	-
$Co(NO_3)_2$ 0,001 mg/L	65.34 ± 4.11%	ab	65.39 ± 6.63%	a	65.36 ± 4.75%	a	44.75 ± 2.74%	a
$Co(NO_3)_2$ 1 mg/L	31.99 ± 1.98%	abcde	33.34 ± 1.86%	abcde	32.42 ± 1.93%	abcd	23.65 ± 1.65%	abc
$Cr(NO_3)_3$ 0,001 mg/L	0.89 ± 0.02%	f	3.31 ± 0.21%	b	1.64 ± 0.06%	e	< LOD	-
$Cr(NO_3)_3$ 1 mg/L	22.20 ± 1.16%	abcdef	22.22 ± 1.38%	abcde	22.21 ± 1.21%	abcde	15.82 ± 0.89%	abc
$Cu(NO_3)_2$ 0.001 mg/L	13.28 ± 0.97%	bcdef	14.94 ± 0.89%	abcde	13.80 ± 0.94%	abcde	8.47 ± 0.70%	bc
$Cu(NO_3)_2$ 1 mg/L	15.37 ± 0.35%	abcdef	57.62 ± 1.17%	ac	28.61 ± 0.54%	abcde	< LOD	-
$Mn(NO_3)_2$ 0.001 mg/L	25.51 ± 2.00%	abcdef	24.94 ± 1.66%	abcde	25.33 ± 1.89%	abcde	17.49 ± 1.51%	abc
$Mn(NO_3)_2$ 1 mg/L	40.77 ± 2.76%	abcd	43.16 ± 4.24%	abcde	41.58 ± 3.34%	abc	32.27 ± 7.80%	ab
$Ni(NO_3)_2$ 0,001 mg/L	23.96 ± 1.95%	abcdef	24.75 ± 1.87%	abcde	24.20 ± 1.92%	abcde	21.34 ± 1.84%	abc
$Ni(NO_3)_2$ 1 mg/L	5.13 ± 0.22%	cdef	12.61 ± 0.20%	abcde	7.48 ± 0.20%	bcde	1.84 ± 0.29%	bc
$Pb(NO_3)_2$ 0.001 mg/L	1.75 ± 0.06%	ef	5.57 ± 0.10%	bde	2.94 ± 0.06%	de	< LOD	-
$Pb(NO_3)_2$ 1 mg/L	40.35 ± 2.87%	abc	42.37 ± 2.53%	acde	40.99 ± 2.76%	ab	29.39 ± 2.33%	ab
$Zn(NO_3)_2$ 0.001 mg/L	0.96 ± 0.11%	f	3.54 ± 0.28%	bd	1.68 ± 0.08%	de	< LOD	-
$Zn(NO_3)_2$ 1 mg/L	2.01 ± 0.11%	def	6.07 ± 0.44%	bcde	3.28 ± 0.18%	cde	0.65 ± 0.10%	c
Positive control	20.53 ± 2.06%	abcdef	63.32 ± 0.86%	ace	32.89 ± 0.90%	abcde	< LOD	-

https://doi.org/10.3390/toxics14090742

Table S4. Reducing sugars, proteins, catalase activity, and guaiacol peroxidase activity expressed as percentages of the negative control (mean ± SD), with compact letter displays (SCLD) indicating statistical differences among treatments. LOD = limit of detection

Tested sample	RS % C-	RS SCLD	P % C-	P SCLD	CAT % C-	CAT SCLD	GPX % C-	GPX SCLD
Negative control	100.00 ± 10.91%	abc	100.00 ± 3.24%	abc	100.00 ± 10.03%	abcd	100.00 ± 12.83%	abc
$Cd(NO_3)_2$ 0,001 mg/L	34.53 ± 2.69%	ab	< LOD	-	12.36 ± 1.43%	fg	34.56 ± 5.06%	bcd
$Cd(NO_3)_2$ 1 mg/L	34.36 ± 2.72%	a	< LOD	-	6.32 ± 1.26%	fg	3.46 ± 0.47%	d
$Co(NO_3)_2$ 0,001 mg/L	132.17 ± 8.07%	abc	65.56 ± 6.10%	ab	77.81 ± 5.60%	bcdef	41.83 ± 2.09%	abcd
$Co(NO_3)_2$ 1 mg/L	131.32 ± 11.31%	abc	3.51 ± 0.98%	a	25.28 ± 1.69%	efg	31.17 ± 2.96%	bcd
$Cr(NO_3)_3$ 0,001 mg/L	131.19 ± 20.96%	abc	129.05 ± 7.47%	abc	128.65 ± 25.34%	abc	49.44 ± 6.13%	abcd
$Cr(NO_3)_3$ 1 mg/L	131.54 ± 9.87%	abc	189.60 ± 9.24%	c	37.08 ± 5.06%	defg	141.53 ± 26.81%	a
$Cu(NO_3)_2$ 0.001 mg/L	132.98 ± 3.42%	abc	162.57 ± 5.95%	bc	48.88 ± 6.74%	bcdef	81.24 ± 7.61%	abc
$Cu(NO_3)_2$ 1 mg/L	48.05 ± 1.29%	ab	< LOD	-	9.27 ± 0.84%	fg	25.56 ± 0.30%	cd
$Mn(NO_3)_2$ 0.001 mg/L	99.47 ± 13.42%	abc	102.12 ± 10.05%	abc	86.38 ± 10.53%	bcdefg	74.57 ± 13.00%	abcd
$Mn(NO_3)_2$ 1 mg/L	76.18 ± 9.23%	ab	115.47 ± 7.58%	abc	64.89 ± 20.22%	defg	84.51 ± 20.24%	abcd
$Ni(NO_3)_2$ 0,001 mg/L	153.24 ± 22.92%	bc	< LOD	-	49.72 ± 6.74%	defg	121.22 ± 22.94%	ab
$Ni(NO_3)_2$ 1 mg/L	110.52 ± 6.72%	abc	24.95 ± 3.30%	a	105.62 ± 7.58%	abcd	64.03 ± 2.80%	abcd
$Pb(NO_3)_2$ 0.001 mg/L	200.79 ± 21.95%	c	76.13 ± 7.37%	abc	53.51 ± 8.01%	cdefg	58.65 ± 1.61%	abcd
$Pb(NO_3)_2$ 1 mg/L	145.96 ± 20.36%	abc	91.34 ± 8.63%	abc	84.27 ± 3.58%	abcde	39.68 ± 4.18%	abcd
$Zn(NO_3)_2$ 0.001 mg/L	75.26 ± 16.64%	abc	97.88 ± 9.36%	abc	172.75 ± 11.80%	a	37.66 ± 3.91%	abcd
$Zn(NO_3)_2$ 1 mg/L	127.81 ± 19.20%	abc	129.88 ± 5.61%	abc	137.08 ± 22.03%	ab	73.46 ± 1.31%	abcd
Positive control	58.39 ± 9.37%	ab	< LOD	-	2.39 ± 0.20%	g	2.56 ± 0.20%	d

Table S5. Reducing sugars, proteins, catalase activity, and guaiacol peroxidase activity normalized to fresh weight and expressed as percentages of the negative control (mean ± SD), with compact letter displays (SCLD) indicating statistical differences among treatments. LOD = limit of detection

Tested sample	RS % C-	RS SCLD	P % C-	P SCLD	CAT % C-	CAT SCLD	GPX % C-	GPX SCLD
Negative control	100.00 ± 10.91%	abc	100.00 ± 3.24%	ab	100.00 ± 10.03%	bcde	115.56 ± 14.83%	bcd
$Cd(NO_3)_2$ 0,001 mg/L	232.90 ± 18.12%	abc	< LOD	-	83.36 ± 9.66%	bcde	269.32 ± 39.43%	a
$Cd(NO_3)_2$ 1 mg/L	395.68 ± 31.36%	ab	< LOD	-	72.79 ± 14.56%	bcde	46.02 ± 6.31%	d
$Co(NO_3)_2$ 0,001 mg/L	145.69 ± 8.89%	abc	72.27 ± 6.73%	a	85.77 ± 6.18%	bcde	53.29 ± 2.66%	d
$Co(NO_3)_2$ 1 mg/L	701.40 ± 60.39%	a	18.72 ± 5.23%	a	135.03 ± 9.00%	bcd	192.35 ± 18.26%	ab
$Cr(NO_3)_3$ 0,001 mg/L	96.08 ± 15.35%	abc	94.52 ± 5.47%	ab	94.22 ± 18.56%	bcde	41.84 ± 5.19%	d
$Cr(NO_3)_3$ 1 mg/L	98.62 ± 7.40%	abc	142.15 ± 6.93%	b	27.80 ± 3.79%	de	122.61 ± 23.23%	bcd
$Cu(NO_3)_2$ 0.001 mg/L	85.33 ± 2.20%	bc	104.32 ± 3.82%	ab	31.36 ± 4.33%	cde	60.24 ± 5.64%	d
$Cu(NO_3)_2$ 1 mg/L	375.03 ± 10.09%	ab	< LOD	-	72.35 ± 6.58%	cde	230.57 ± 2.73%	ab
$Mn(NO_3)_2$ 0.001 mg/L	95.70 ± 12.91%	abc	98.25 ± 9.67%	ab	83.10 ± 10.13%	bcde	82.91 ± 14.45%	d
$Mn(NO_3)_2$ 1 mg/L	71.49 ± 8.66%	c	108.37 ± 7.12%	ab	60.90 ± 18.98%	cde	91.64 ± 21.95%	cd
$Ni(NO_3)_2$ 0,001 mg/L	102.25 ± 15.30%	abc	< LOD	-	33.17 ± 4.50%	e	93.47 ± 17.69%	cd
$Ni(NO_3)_2$ 1 mg/L	320.63 ± 19.50%	abc	72.37 ± 9.56%	a	306.41 ± 21.99%	a	214.65 ± 9.37%	abc
$Pb(NO_3)_2$ 0.001 mg/L	236.74 ± 25.88%	abc	89.77 ± 8.69%	ab	63.09 ± 9.44%	bcde	79.91 ± 2.19%	cd
$Pb(NO_3)_2$ 1 mg/L	137.04 ± 19.12%	abc	85.76 ± 8.10%	ab	79.12 ± 3.36%	bcde	43.05 ± 4.53%	d
$Zn(NO_3)_2$ 0.001 mg/L	59.13 ± 13.07%	c	76.91 ± 7.36%	ab	135.74 ± 9.27%	bc	34.19 ± 3.55%	d
$Zn(NO_3)_2$ 1 mg/L	134.23 ± 20.16%	abc	136.41 ± 5.90%	b	143.97 ± 23.14%	b	89.15 ± 1.59%	cd
Positive control	685.34 ± 109.92%	a	< LOD	-	28.02 ± 2.33%	e	34.72 ± 2.74%	d

References

1. Bian, J.; Berninger, J.P.; Fulton, B.A.; Brooks, B.W. Nutrient stoichiometry and concentrations influence silver toxicity in the aquatic macrophyte *Lemna gibba*. *Sci Total Environ* **2013**, *449*, 229–236, doi:https://doi.org/10.1016/j.scitotenv.2012.12.098
2. Lee, H.; De Saeger, J.; Bae, S.; Kim, M.; Depuydt, S.; Heynderickx, P.M.; Wu, D.; Han, T.; Park, J. Giant duckweed (*Spirodela polyrhiza*) root growth as a simple and sensitive indicator of copper and chromium contamination. *Toxics* **2023**, *11*, 788, doi:https://doi.org/10.3390/toxics11090788
3. Pereira, S.P.P.; Jesus, F.; Aguiar, S.; de Oliveira, R.; Fernandes, M.; Ranville, J.; Nogueira, A.J.A. Phytotoxicity of silver nanoparticles to *Lemna minor*: Surface coating and exposure period-related effects. *Sci Total Environ* **2018**, *618*, 1389–1399, doi:https://doi.org/10.1016/j.scitotenv.2017.09.275

4. Naumann, B.; Eberius, M.; Appenroth, K.-J. Growth rate based dose–response relationships and EC-values of ten heavy metals using the duckweed growth inhibition test (ISO 20079) with *Lemna minor* L. clone St. *J Plant Physiol* **2007**, *164*, 1656–1664, doi:https://doi.org/10.1016/j.jplph.2006.10.011
5. Rahman, M.A.; Hogan, B.; Duncan, E.; Doyle, C.; Krassoi, R.; Rahman, M.M.; Naidu, R.; Lim, R.P.; Maher, W.; Hassler, C. Toxicity of arsenic species to three freshwater organisms and biotransformation of inorganic arsenic by freshwater phytoplankton (*Chlorella sp.* CE-35). *Ecotox Environ Safe* **2014**, *106*, 126–135, doi:https://doi.org/10.1016/j.ecoenv.2014.03.004
6. Horemans, N.; Van Hees, M.; Van Hoeck, A.; Saenen, E.; De Meutter, T.; Nauts, R.; Blust, R.; Vandenhove, H. Uranium and cadmium provoke different oxidative stress responses in *Lemna minor* L. *Plant Biol* **2015**, *17*, 91–100, doi:https://doi.org/10.1111/plb.12222
7. Khellaf, N.; Zerdaoui, M. Growth response of the duckweed *Lemna minor* to heavy metal pollution. *Iran. J. Environ. Health. Sci. Eng.* **2010**, *6*, 161–166, https://utoronto.scholaris.ca/server/api/core/bitstreams/cc435383-99a0-4bfe-a279-04221e0f6d85/content.
8. Modlitbová, P.; Novotný, K.; Pořízka, P.; Klus, J.; Lubal, P.; Zlámalová-Gargošová, H.; Kaiser, J. Comparative investigation of toxicity and bioaccumulation of Cd-based quantum dots and Cd salt in freshwater plant *Lemna minor* L. *Ecotox Environ Safe* **2018**, *147*, 334–341, doi:https://doi.org/10.1016/j.ecoenv.2017.08.053
9. Basile, A.; Sorbo, S.; Conte, B.; Cobianchi, R.C.; Trinchella, F.; Capasso, C.; Carginale, V. Toxicity, accumulation, and removal of heavy metals by three aquatic macrophytes. *Int J Phytoremediat* **2012**, *14*, 374–387, doi:https://doi.org/10.1080/15226514.2011.620653
10. Drost, W.; Matzke, M.; Backhaus, T. Heavy metal toxicity to *Lemna minor:* studies on the time dependence of growth inhibition and the recovery after exposure. *Chemosphere* **2007**, *67*, 36–43, doi:https://doi.org/10.1016/j.chemosphere.2006.10.018
11. Stubblefield, W.A.; Van Genderen, E.; Cardwell, A.S.; Heijerick, D.G.; Janssen, C.R.; De Schamphelaere, K.A.C. Acute and chronic toxicity of cobalt to freshwater organisms: Using a species sensitivity distribution approach to establish international water quality standards. *Environ Toxicol Chem* **2020**, *39*, 799–811, doi:https://doi.org/10.1002/etc.4662
12. Song, L.; Vijver, M.G.; Peijnenburg, W.J.G.M. Comparative toxicity of copper nanoparticles across three Lemnaceae species. *Sci Total Environ* **2015**, *518-519*, 217–224, doi:https://doi.org/10.1016/j.scitotenv.2015.02.079
13. Vulpe, C.-B.; Bulancea, C.-C.; Doczi, G.G.; Roman, D.-L.; Agachi, B.-V. Ecotoxicological impact of copper and manganese salts on *Lemna minor*: Growth inhibition and biochemical alterations. *Ann West Univ Tim Ser Bio* **2025**, *28*, 17–26, https://cbg.uvt.ro/wp-content/uploads/2025/08/AWUTSerBio_July2025_17-26_Vulpe_Agachi.pdf.
14. Charles, A.L.; Markich, S.J.; Ralph, P. Toxicity of uranium and copper individually, and in combination, to a tropical freshwater macrophyte (*Lemna aequinoctialis*). *Chemosphere* **2006**, *62*, 1224–1233, doi:https://doi.org/10.1016/j.chemosphere.2005.04.089
15. Roubeau Dumont, E.; Larue, C.; Lorber, S.; Gryta, H.; Billoir, E.; Gross, E.M.; Elger, A. Does intraspecific variability matter in ecological risk assessment? Investigation of genotypic variations in three macrophyte species exposed to copper. *Aquat Toxicol* **2019**, *211*, 29–37, doi:https://doi.org/10.1016/j.aquatox.2019.03.012
16. Rodrigues, A.C.M.; Jesus, F.T.; Fernandes, M.A.F.; Morgado, F.; Soares, A.M.V.M.; Abreu, S.N. Mercury toxicity to freshwater organisms: Extrapolation using species sensitivity distribution. *B Environ Contam Tox* **2013**, *91*, 191–196, doi:https://doi.org/10.1007/s00128-013-1029-0
17. Subhadra, A.V.; Nanda, A.K.; Behera, P.K.; Panda, B.B. Acceleration of catalase and peroxidase activities in *Lemna minor* L. and *Allium cepa* L. in response to low levels of aquatic mercury. *Environ Pollut* **1991**, *69*, 169–179, doi:https://doi.org/10.1016/0269-7491(91)90141-I
18. Dirilgen, N. Mercury and lead: Assessing the toxic effects on growth and metal accumulation by *Lemna minor*. *Ecotox Environ Safe* **2011**, *74*, 48–54, doi:https://doi.org/10.1016/j.ecoenv.2010.09.014
19. Lahive, E.; O' Halloran, J.; Jansen, M.A.K. Differential sensitivity of four Lemnaceae species to zinc sulphate. *Environ Exp Bot* **2011**, *71*, 25–33, doi:https://doi.org/10.1016/j.envexpbot.2010.10.014

Disclaimer/Publisher's Note: The statements, opinions and data contained in all publications are solely those of the individual author(s) and contributor(s) and not of MDPI and/or the editor(s). MDPI and/or the editor(s) disclaim responsibility for any injury to people or property resulting from any ideas, methods, instructions or products referred to in the content.
